# Findings from the Hispanic Community Health Study/Study of Latinos on the Importance of Sociocultural Environmental Interactors: Polygenic Risk Score-by-Immigration and Dietary Interactions

**DOI:** 10.3389/fgene.2021.720750

**Published:** 2021-12-06

**Authors:** Cristin E. McArdle, Hassan Bokhari, Clinton C. Rodell, Victoria Buchanan, Liana K. Preudhomme, Carmen R. Isasi, Mariaelisa Graff, Kari North, Linda C. Gallo, Amber Pirzada, Martha L. Daviglus, Genevieve Wojcik, Jianwen Cai, Krista Perreira, Lindsay Fernandez-Rhodes

**Affiliations:** ^1^ Department of Biobehavioral Health, College of Health and Human Development, The Pennsylvania State University, University Park, PA, United States; ^2^ Carey Business School, Johns Hopkins University, Baltimore, MD, United States; ^3^ Department of Epidemiology, Gillings School of Global Public Health, University of North Carolina at Chapel Hill, Chapel Hill, NC, United States; ^4^ Department of Psychology, University of Miami, Coral Gables, FL, United States; ^5^ Department of Epidemiology and Population Health, Albert Einstein College of Medicine, Bronx, NY, United States; ^6^ Carolina Center for Genome Sciences, School of Medicine, University of North Carolina at Chapel Hill, Chapel Hill, NC, United States; ^7^ Department of Psychology, San Diego State University, San Diego, CA, United States; ^8^ Institute for Minority Health Research, Carle Illinois College of Medicine, University of Illinois at Urbana–Champaign, Champaign, IL, United States; ^9^ Institute for Minority Health Research, University of Illinois at Chicago, Chicago, IL, United States; ^10^ Department of Epidemiology, Johns Hopkins University, Baltimore, MD, United States; ^11^ Department of Biostatistics, Gillings School of Global Public Health, University of North Carolina at Chapel Hill, Chapel Hill, NC, United States; ^12^ Department of Social Medicine, University of North Carolina School of Medicine, Chapel Hill, NC, United States

**Keywords:** polygenic risk score, gene-environment interactions, Hispanic/Latino, acculturation, obesity

## Abstract

**Introduction:** Hispanic/Latinos experience a disproportionate burden of obesity. Acculturation to US obesogenic diet and practices may lead to an exacerbation of innate genetic susceptibility. We examined the role of gene–environment interactions to better characterize the sociocultural environmental determinants and their genome-scale interactions, which may contribute to missing heritability of obesity. We utilized polygenic risk scores (PRSs) for body mass index (BMI) to perform analyses of PRS-by-acculturation and other environmental interactors among self-identified Hispanic/Latino adults from the Hispanic Community Health Study/Study of Latinos (HCHS/SOL).

**Methods:** PRSs were derived using genome-wide association study (GWAS) weights from a publicly available, large meta-analysis of European ancestry samples. Generalized linear models were run using a set of *a priori* acculturation-related and environmental factors measured at visit 1 (2008–2011) and visit 2 (2014–2016) in an analytic subsample of 8,109 unrelated individuals with genotypic, phenotypic, and complete case data at both visits. We evaluated continuous measures of BMI and waist-to-hip ratio. All models were weighted for complex sampling design, combined, and sex-stratified.

**Results:** Overall, we observed a consistent increase of BMI with greater PRS across both visits. We found the best-fitting model adjusted for top five principal components of ancestry, sex, age, study site, Hispanic/Latino background genetic ancestry group, sociocultural factors and PRS interactions with age at immigration, years since first arrival to the United States (*p* < 0.0104), and healthy diet (*p* < 0.0036) and explained 16% of the variation in BMI. For every 1-SD increase in PRS, there was a corresponding 1.10 kg/m^2^ increase in BMI (*p* < 0.001). When these results were stratified by sex, we observed that this 1-SD effect of PRS on BMI was greater for women than men (1.45 vs. 0.79 kg/m^2^, *p* < 0.001).

**Discussion:** We observe that age at immigration and the adoption of certain dietary patterns may play a significant role in modifying the effect of genetic risk on obesity. Careful consideration of sociocultural and immigration-related factors should be evaluated. The role of nongenetic factors, including the social environment, should not be overlooked when describing the performance of PRS or for promoting population health in understudied populations in genomics.

## Introduction

Obesity is a significant public health issue, with an estimated 34.9% of adults and 16.9% children in the United States reported as obese (Ogden et al., 2014). From 1999 to 2018, the prevalence of both obesity and severe obesity continued to increase among adults (Hales et al., 2020). By 2030, the economic consequences of obesity if left unaddressed could represent up to $66 billion per year in the United States (Wang et al., 2011) and a concomitant increase in health sequelae, most notably cardiovascular disease (CVD), diabetes, cancer, and loss of quality-adjusted life years (National Heart, Lung, and Blood Institute 2013). Hispanic/Latino adults bear a disproportionate burden of obesity, as they are 1.2 times more likely to be obese than non-Hispanic Whites (OMH, 2020). The age-adjusted prevalence of obesity is approximately 42.6% obesity Hispanic adults in the United States (as compared with 37.7% among non-Hispanic White adults) (Flegal et al., 2012). Additionally, there are observed gender disparities, wherein women have a higher prevalence of obesity than men, related, in part, to a complex set of sociocultural and other environmental factors or biologic sex-related differences (Kanter and Caballero 2012; Link and Reue 2017).

Extensive evidence supports the role of lifestyle modification for reducing obesity; however, less attention has been paid to understanding the role of gene–environment (G×E) interactions in population trends or when informing precision medicine initiatives. G×E interactions are believed to play an important role in obesity (Flowers et al., 2012; Ahmad et al., 2013), as they have the potential to inform our understanding of how environmental determinants affect an individual’s health. In fact, G×E studies can lead to a more accurate genetic effect estimation than that observed in the original discovery study and may account in part for “missing heritability” of complex traits, like obesity (Flowers et al., 2012). Obesogenic environments, such as those in the United States, may differentially (Ogden et al., 2007) impact individuals who carry more genetic susceptibility variants. Environmental interactions with a polygenic score for obesity have been previously reported for sugar-sweetened beverages (Qi et al., 2012), dietary patterns, specifically fried food (Qi et al., 2014), calorie (Lee et al., 2021) and alcohol intake (Nakamura et al., 2016), and physical activity (Tyrrell et al., 2017). These PRS–environment interactions informed our *a priori* selection of environmental factors to evaluate. Yet to our knowledge, limited work has been done to date to also investigate how acculturation—a complex process of an individual’s “maintenance of the original cultural and development of relationships with the new (host) culture” (Berry 2004; Thomson and Hoffman-Goetz 2009; Wallace et al., 2010)—may modify the observed effect of established genetic risk for obesity, given its relevance in the social sciences and public health (Khan et al., 1997; Sundquist and Winkleby 2000; Gordon-Larsen et al., 2003; Barcenas et al., 2007; Pérez-Escamilla and Putnik 2007; Duffey et al., 2008; Roshania et al., 2008; Guendelman et al., 2011; Van Wieren et al., 2011; Liu J.-H. et al., 2012; Murillo et al., 2015).

G×E effects on measures of obesity may be assessed using any of the three following approaches (Nagpal et al., 2018): by measuring 1) individual genotypes or single-nucleotide polymorphisms (SNPs), 2) polygenic genetic liability of disease, and 3) whole-genome common variants (SNP heritability). Herein, we build on a previous study in Hispanic Community Health Study/Study of Latinos (HCHS/SOL) that reported interactions between genetic risk score across 103 SNPs and acculturation, wherein the observed genetic effects were the highest among the most acculturated and among women, as compared with the least acculturated and men (under review at *Demography*).

In this current study, we aimed to further investigate the G×E interaction by using a more encompassing predictor of disease risk—the polygenic risk score (PRS)—and by taking a more comprehensive view of the environment. PRSs allow for a single measure of genetic liability for disease risk or inherited obesity susceptibility (Khera et al., 2019). PRSs are commonly constructed as the weighted sum of scores using effects sizes from genome-wide association studies (GWASs) as their weights and the count of observed risk allele and linkage disequilibrium based on the dataset of interest (Grinde et al., 2019). Increasingly, there is evidence that obesity is highly polygenic (Choquet and Meyre 2011; Reddon et al., 2016); and to date, over 1,000 unique obesity-related genome wide association study loci have been identified (GWAS Catalog, 2020). Yet most G×E interaction analyses to date of obesity have been limited to <100 genetic variants accounting for <3% variation in body mass index (BMI) (Locke et al., 2015; Reddon et al., 2016; Grinde et al., 2019).

Overall, we sought to investigate the association between a robust measure of genetic susceptibility to BMI (PRS_BMI_) and BMI and evidence of effect modification by acculturation, immigration, lifestyle, and other environmental factors in a diverse community-based sample of Hispanic/Latino adults. We described the utility of the PRS_BMI_ prior to estimating the G×E interactions with respect to acculturation, immigration, lifestyle, and other environmental factors. We then evaluated the stability of these genetic estimates over time by examining the role of PRS_BMI_, and G×E interactions at baseline and a 6-years follow-up. We hypothesized that acculturation to the United States may be associated with a number of adaptations to a new host culture that could lead to an additional level of modification of innate genetic susceptibility in the Hispanic/Latino community. We hypothesized that greater acculturation to the United States would exacerbate the PRS association with elevated BMI, obesity, or greater adulthood change in BMI, consistent with a diathesis-stress gene–environment interaction model (Boardman et al., 2014). We tested this hypothesis with genetic (PRS_BMI_) and BMI information as well as measures of acculturation using a sample of 8,109 US Hispanic/Latino adults aged 20–76 years from four HCHS/SOL urban communities (The Bronx, NY; Chicago, IL; Miami, FL; and San Diego, CA).

## Methods

### Study Population

The HCHS/SOL is cohort of 16,415 individuals aged 18–74 years in 2008–2011 from four US urban communities and diverse cultural and genetic origins who self-identified as being Hispanic/Latino. Individuals in the HCHS/SOL provided information about their background (heritage) as being of Cuban, Dominican, Puerto Rican, Mexican, Central American, South American, or other/multiple backgrounds. Recruitment for the HCHS/SOL was implemented through a two-stage area household probability design (Lavange et al., 2010). Study individuals completed visit 1 (2008–2011) and visit 2 (2014–2017). As previously reported, HCHS/SOL individuals provided informed consent for genetic testing at visit 1 and passed quality control measures (Sofer et al., 2018), 10,240 of whom were unrelated (defined as second-degree or beyond) first and included in PRS estimation, described below. Complete case analysis based on non-missing values for visit 1 and visit 2 was performed on a subset of individuals with genotype and phenotype information (see Supplementary Figure S1 for inclusion/exclusion flowchart). These exclusions decreased the available analytic sample in HCHS/SOL to 8,109 Hispanic/Latino adults, aged 20–76 years at visit 1 and 6,408 for visit 2. The HCHS/SOL was approved by each site’s local institutional review board as well as at the Coordinating Center.

### Genotyping, Quality Control, and Examination Methods

#### Genotyping

The HCHS/SOL study protocol and examination, genotyping, quality control, and imputation methods have been described previously (Conomos et al., 2016; Sofer et al., 2018). Briefly, DNA was extracted from individual blood samples and genotyped on the MEGA Custom 15041502 array (Illumina Omni2.5M + custom content). Quality control measures included constructing principal components (PCs) of genetic ancestry to account for population stratification. Imputation was then conducted to 1000 Genomes Project phase 3 based on 1000 Genomes Project reference populations.

#### Polygenic Risk Score Estimation

The PRS_BMI_ was estimated to capture genome-wide risk of elevated BMI for subsequent G×E analyses, using HCHS/SOL genome-wide data and publicly available genome-wide effect sizes from a recent European ancestry meta-analysis of ∼700,000 GIANT and United Kingdom Biobank (UKBB) samples (Yengo et al., 2018). PRS was calculated for SNPs with minor allele frequency >0.5% in HCHS/SOL based on effect sizes from the GWAS of Yengo et al. (2018) using the LDpred method and infinitesimal model described previously (Vilhjálmsson et al., 2015). The best-fitting PRS from the LDpred method predicted 7.4% of the variance in inverse normalized BMI in HCHS/SOL after accounting for age, sex, the first 10 PCs, genetic ancestry background Hispanic/Latino group, and the normalized log of the sample weight. This best-fitting PRS was then standardized to a mean of 0 and standard deviation of 1 and carried forward to our G×E analyses described in detail below, although most results are reported as a 1-SD change in PRS from the mean. In sensitivity analyses, we additionally considered a decile change in PRS_BMI_ to be consistent with other published reports (Khera et al., 2019).

#### Obesity-Related Measurements

BMI was calculated as the ratio of an individual’s weight (kg) to their height (m) squared. Both weight and height were measured at examination by trained staff following a standardized protocol in HCHS/SOL (Sorlie et al., 2010). Similar to previous analyses of genetic variation in HCHS/SOL samples (Fesinmeyer et al., 2013; Gong et al., 2018), we excluded individuals <20 years of age or with BMIs <18.5 or >70.0 kg/m^2^ from our analytic sample, to focus on individuals who had both completed growing and were unlikely to have monogenic forms of obesity or gross data errors (Fesinmeyer et al., 2013; Gong et al., 2018). The resulting beta coefficients can be interpreted as change in kg/m^2^ of BMI for a 1-SD change from the mean PRS_BMI_, or per decile (10%) change from the median in our sensitivity analyses. In addition, we examined alternate specifications for adiposity including a dichotomous measure of obesity (as a BMI ≥ 30 kg/m^2^) and a continuous waist-to-hip ratio.

#### Sociocultural Environments: Acculturation- and Immigration-Related Measures

Acculturation is a multidimensional, directional, and dynamic process, which is heavily influenced by both the timing and pattern of sociocultural exposures across one’s life course (Ben-Shlomo and Kuh 2002; Berry 2004; Wallace et al., 2010). Proxy measures of acculturation, such as language preference, nativity, and time in the United States, are commonly collected in large epidemiologic studies (Thomson and Hoffman-Goetz 2009). Although the ability of such proxies to capture the complexity of the construct of acculturation has been criticized (Carter-Pokras and Bethune 2009; Thomson and Hoffman-Goetz 2009), they remain a staple of epidemiologic research due to their accessibility. The baseline HCHS/SOL visit 1 was conducted in either English or Spanish based on the individual’s preference and included questionnaires about an individual’s immigration history (e.g., nativity of one’s self and their family members, and information about first arrival to the United States), self-identified Hispanic/Latino background, and 10-item modification of the Short Acculturation Scale for Hispanics scale (Marin et al., 1987), a uni-dimensional/uni-directional scale that includes Language Use and Ethnic and Social & Ethnic Relations, each ranging from 1 to 5 low-to-high acculturation. Language preference was the spoken language preferred by the participant at visit 1 (Spanish or English).

In addition to the above measures, five categories of age at immigration (US-born, 0–5, 6–12, 13–20, and ≥21 years) were constructed using nativity status, and time since first arrival to measure the duration of exposure to the obesogenic US culture, consistent with other works using a sensitive period of acculturation (Rumbaut 2005) that evaluates the age at immigration. We categorized this new variable as US-born, 0–5, 6–12, 13–20, and ≥21 years and for simplicity refer to it as an “immigration-related” sociocultural factor, even though it is composed of multiple proxies of acculturation (described above). In addition to this sensitive period of acculturation, immigrant generation status may be an important predictor of duration or pattern of sociocultural exposures (Chrisman et al., 2017) and was also included in our modeling. Thus, in order to understand these migration and acculturation patterns, we examined immigrant generation status (first or second) based on a combination of the individual’s country of origin, maternal and paternal parents, and maternal and grandparents as first generation, which includes foreign-born with foreign-born parents including those born in a US territory, compared with second generation, which includes US-born (50 states and DC) or foreign-born with at least one US-born parent.

We hypothesized that the more adapted an individual is to the host culture (e.g., US-dominant culture), the greater the risk for elevated BMI or obesity. In particular, we expected that this adaptation might be strongest for those who were the youngest at first arrival to the United States (Roshania et al., 2008; Rumbaut 2005; Stevens 1999), individuals of second-generation immigrant status (e.g., US-born to at least one immigrant parent) (Liu J.-H. et al., 2012; Chrisman et al., 2017), and those with a strong English-language (Chrisman et al., 2017) preference.

#### Additional Environmental Factors

We evaluated additional environmental measures of sociodemographic, chronic disease, and lifestyle factors in our G×E modeling based on previous literature (Qi et al., 2012; 2014; Lee et al., 2021; Nakamura et al., 2016; Tyrrell et al., 2017; Qi et al., 2015). These measures included the following: sex (male, female); age and age^2^ to account for nonlinear changes in BMI with age; genetic ancestry background Hispanic/Latino group (Central American, Cuban, Dominican, Puerto Rican, South American, and Mexican) (Conomos et al., 2016); marital status (married and living with partner, or not); education (less than high school, or completed high school or greater); employment status (retired, not retired or employed, employed ≤ 35 h/week, employed > 35 h/week); diabetes status (yes, no); prevalent CVD (yes, no); sleep duration (h/day); consumption of sweetened beverages (servings/day); physical activity guidelines met (yes/no); alcohol use level (no current use, low-level use, and high-level use); cigarette use (never, current, and former); Center for Epidemiological Studies Depression (CES-D) 10-item Summary Score (Andresen et al., 1994); and ethnic identity score (5 level ordinal variable). We independently evaluated two overall measures of diet derived from the 24-h dietary recalls collected as a part of visit 1 to better understand a diet-acculturation convention that immigrants consumed healthier diets before migration (Martínez 2013). The first score was a dietary measure published in the *Journal of the American Medical Association* (JAMA) (Daviglus et al., 2012) and calculated by assigning individuals a score of 1–5 (low = 1 to high = 5) according to sex-specific quintiles of usual predicted daily intake of saturated fatty acids, potassium, calcium, and fiber. The highest 40% on the summed score was defined as a healthy diet (Liu K. et al., 2012). A second score, the Alternate Healthy Eating Index 2010 (AHEI-2010), is a measure of diet quality based on foods and nutrients predictive of chronic disease risk (Chiuve et al., 2012). AHEI-2010 score is the sum of the 11 individual components’ scores, each with a range from 0 (worst) to 10 (best). Hence, AHEI-2010 healthy dietary measure takes values from 0 to 110, where higher scores represent healthy eating habits. In sensitivity analysis, we evaluated information specific to dietary acculturation as self-identified dietary ranking as more American or more Hispanic on a scale of 1–5.

### Analysis

We first examined the robustness of our PRS_BMI_ in our target population by describing its distribution, calibration, discrimination, and predictive ability, consistent with recent PRS reporting standards (Wand et al., 2021). We examined the adjusted r^2^ for models with basic covariates with and without the PRS_BMI_ to assess the incremental variation to ensure that the PRS_BMI_ would be a robust predictor for use in additional G×E analyses.

In our inferential modeling, using survey linear regression, we tested the association of BMI and PRS_BMI_ × acculturation using data from visit 1 and 2. Continuous predictors were survey mean-centered and standardized to avoid multicollinearity in interaction models. Our full modeling used augmented backwards elimination that included sample weights (Pfeffermann 1996) to increase the chance of a reliable outcome that recovered our underlying target population (Heeringa et al., 2017; Pfeffermann 1996; Dunkler et al., 2014; Heinze et al., 2018) with passive explanatory variables, not tested for significance or change in criterion, which included the PRS_BMI_ and the top five PCs, study center, and Hispanic/Latino background. We performed a three-stage model building, first examining linear regression main effects by augmented backwards elimination, then G×E interaction modeling separately, and finally a fully adjusted model with both main and interaction effects based on backwards elimination (see Supplementary Figure S2 for modeling diagram and Supplementary Table S1 for variable and modeling descriptions). In stage 1 of model building, we performed augmented backwards elimination starting with a global model that included passive variable (PRS, top five PCs, study center, and Hispanic/Latino background) and active variables, based on our *a priori* covariates (sociocultural, immigration-related, acculturation, and additional environmental factors) that were conceptually relevant. We assessed that the global model was fitted by the weighted least squares and used a progressive significance level of *p* < 0.20 or change in criterion tau 0.05, until all variables contributed to the model and were retained. Stage 2 testing included interaction modeling of all the previous variables tested adjusted for passive variables and interacted with PRS. We assessed joint interaction effects for nominal significance (*p* < 0.05). We tested the null hypothesis that the H_0_: β G×E = 0 retaining those interactions with two-sided t-test at significance level *α* = 0.05 and then retained interactions in Stage 3, the fully adjusted model linear regression modeling with interaction variables, based on Bonferroni significance adjustment (0.05/4 = 0.0125) for multiple comparisons. Parameter estimates for our PRS_BMI_ are reported with Taylor series linearization variance estimator for the full model and sex-stratified.

Linear regression modeling was used to estimate the G×E interactions (PRS_BMI_ × E) on BMI for the above-specified sociocultural environmental and additional environmental factors (E) at visit 1. All interaction models took the form of

$$y_{BMI\;\;}=\;\beta_{0}+\beta_{G}G+\;\beta_{Ei}E_{i}\;+\beta_{GxEi}G*E_{i}+\;\beta_{cov1}x_{Cov1}+\;+\varepsilon$$ (1)

where G is the genetic risk of obesity as measured by the PRS for BMI, PRS_BMI_; E_i_ is environmental (environmental sociocultural factors); X_cov1_ is the vector of top five PCs; X_cov2_ is the vector of covariates age, age^2^; $\beta_{0}$ is the intercept; $\beta_{G}$ is the PRS main effect; $\beta_{Ei}$ is the environmental effect from acculturation or sociocultural factor; the gene–environment effect is $\beta_{GxEi}G*E_{i}$ ; $\beta_{cov1}$ are passive variables (top five PC effects, study center, Hispanic/Latino background group); and ε is the error term.

We then repeated our assessment of interaction for visit 2 to better evaluate those interactions most and least sensitive to age-related BMI changes.

The base model includes adjustment for the top five PCs, study center, and genetic Hispanic/Latino ancestry. The full model includes the base model and age, age^2^, sex, age at immigration, immigrant generation, sleep duration, meets physical activity guidelines, sweetened beverage consumption, JAMA healthy diet, alcohol use level, cigarette use, CES-D 10-item Summary Score, employment status, income, diabetes and CVD prevalence, $\beta_{GxE}$ for age at immigration, and $\beta_{GxE}$ for JAMA healthy diet. For models with interactions $\beta_{GEi}$ , the main effect of β_G_, which represents PRS_BMI_, are conditional on interacting with that predictor. We plotted effects of the significant interaction terms graphically. Survey commands were used in SAS 9.4 (Cary, NC, United States) for the weighted backwards elimination model choice and estimation. SAS code is available upon request.

In an exploratory analysis, we further examined our results using an alternative specification for dietary measurement, the AHEI-2010 given that it may be a more culturally appropriate measure of diet quality for this population by measuring a more diverse selection of food choices (Chiuve et al., 2012). Finally, we also examined an incremental model selection stratified by genetic ancestry Hispanic/Latino background group to better understand how genetic ancestry might influence PRS performance and model selection.

## Results

In our complete case analytic dataset of 8,109 Hispanic/Latino adults, we examined the variable distributions using survey univariate analysis for visit 1 (Table 1) and reported the weighted means and proportions (see Supplementary Table S2 for target population and for visit 2 demographic characteristics). Overall, the weighted sample was 48.9% female, 41.7% considered to be obese, with 65.3% with an annual income of <$30,000, 54.1% being currently employed at least part time, 69.5% with at least a high school education or equivalent, 56.3% currently married or living with a partner, 16.4% living with type 2 diabetes, and 6.6% living with prevalent CVD, such as cardiovascular heart disease or stroke. Men and women had similar weighted average age at visit 1, scores for acculturation subscales for Language Use and Social & Ethnic Relations (2.1–2.2), and distributions of age at immigration. Although nearly 20.4% of male and 17.1% of female individuals were born to US- and foreign-born individuals who have lived in the United States for an average of 17.8 years, the majority arrived during adulthood (53.8% of males and 57.2% of females). Dietary behaviors differed across the sexes, with males consuming more sweetened beverage servings daily (mean 2.2 compared with 1.3 for females) but with similar Healthy Diet scores by sex, with 48.6% males and 47.5% female in the top 40th percentile of the JAMA dietary value.

**TABLE 1:** Demographic characteristics Hispanic Community Health Study/Study of Latinos (HCHS/SOL) analytic subsample from visit 1 (unweighted *n* = 8,109), overall and sex-stratified.

	Weighted total		Male		Female	
	N or Mean	% or SE	N or Mean	% or SE	N or Mean	% or SE
Total	8.109	100	3,467	51.1	4,642	48.9
Weighted	7,791		3,981		3,810	
Age (years)	44	0.3	43.7	0.4	44.6	0.4
Study center						
The Bronx	2,215	28.4	1,030	25.9	1,186	31.1
Chicago	1,170	15.0	633.1	15.9	536.7	14.1
Miami	2,429	31.1	1,295	32.5	1,134	29.8
San Diego	1,977	25.4	1,023	25.7	953.8	25.0
Background*						
Central American	638.1	8.2	330	8.3	308	8.1
Cuban	1,847	23.7	1,020	25.6	827.6	21.7
Dominican	711.3	9.1	292.5	7.3	418.8	11.0
Mexican	2,823	36.2	1,430	35.9	1,393	36.6
Puerto Rican	1,329	17.1	692	17.4	637.2	16.7
South American	442	5.7	216	5.4	226	5.9
Acculturation/Nativity Measures						
Born in the United States	1,465	18.8	813.5	20.4	652	17.1
Years lived in the United States	17.8	0.37	17.6	0.43	18.1	0.47
Age at immigration*						
Born in United States	1,465	18.8	813.5	20.4	652	17.1
(0 to <18)	364.9	4.7	165.2	4.1	199.7	5.2
(6–12)	363.8	4.7	174.2	4.4	189.6	5.0
(13–20)	1,274.0	16.3	685.2	17.2	588.5	15.4
(21+)	4,323	55.5	2,143.0	53.8	2,181	57.2
Period of immigration						
Born in the US	1,547	19.5	859	21.2	687	17.8
Before 1980	5,305	66.2	2,936	65.0	2,604	67.4
After 1980	1,616	14.3	660	13.8	572	14.8
First-generation immigrant*	6,207	79.7	3,115	8.2	3,092	81.2
SASH-Lang	2.0	0.0	2.2	0.0	1.9	0.0
SASH-Soc	2.2	0.0	2.3	0.0	2.2	0.0
Sociocultural measures						
Education*						
Less than HS	2,394	30.5	1,183	29.7	1,211	31.8
At least HS or equiv	5,397	69.5	2,798	70.3	2,599	68.2
Married, living with spouse*	4,390	56.3	2,390	60.0	2,000	52.5
Household income*						
Less than $30,000	5,104	65.5	2,416	60.7	2,687	70.5
At least $30,000 USD	2,688	34.5	1,564	39.3	1,123	29.5
4-Level employment status*						
Retired and not currently working	728.4	9.3	371.7	9.3	356.7	9.2
Not retired and not currently working	2,853	36.6	1,133	28.5	1,720	45.1
Employed ≤ 35 h/week	1,290	16.6	569	14.3	720.8	18.9
Employed > 35 h/week	2,920	37.5	1,907	47.9	1,013	26.6
Ethnic Identity Summary Score	3.2	0.0	3.2	0.0	3.2	0.0
CES-D 10-item Summary Score	6.9	0.1	6.0	0.0	8.0	0.0
Sweetened beverage consumption Servings/day	1.8	0.0	2.2	0.0	1.3	0.0
Meets 2008 Physical Health Guidelines*	5,185	66.6	2,963	74.4	2,223	58.3
Healthy diet (top 40% for sex)	3,746	8.1	1,936	48.6	1,810	47.5
Average sleep duration (h/day)	7.9	0.02	7.8	0.0	8.0	0.0
Cigarette use						
Never	4,637	59.5	1964	49.3	2673	70.1
Former	1,512	19.4	1023	25.7	488.3	12.8
Current	1,642	21.1	992.8	24.9	649.2	17.0
Alcohol use*						
Non-drinker	3,711	47.6	1,500	37.7	2,221	58.0
Low-risk drinker	3,594	46.1	2,131	53.5	1,463	38.4
At risk drinker	486.2	6.2	349.3	8.8	136	3.6
Diabetes history						
No	6,517	83.6	3,333	83.7	3,184	83.6
Yes	1,274	16.4	648	16.3	627	16.4
CVD risk factors*						
No	7,280	93.4	3,671	92.2	3,609	94.7
Yes	512	6.6	309.5	7.8	201.7	5.3
Measures of adiposity						
BMI (kg/m^2^)	29.8	0.09	29.2	0.1	30.4	0.1
Waist-to-hip ratio	0.9	0.0	1.0	0.0	0.9	0.0
Obesity						
<BMI 30	4,542	58.3	2,443	61.4	2,098	55.1
≥BMI 30	3,249	41.7	1,537	38.6	1,712	44.9

*p < 0.05 total: Rao–Scott X^2^ tests for categorical variable, t-test for mean.

BMI, body mass index; PRS, polygenic risk score; SASH-Lang, SASH Language Subscale; SASH-Soc, SASH Social and Ethnic Relations Subscale; CVD, cardiovascular disease.

Note. Normal, 18.5 < BMI < 25; overweight, 25 ≤ BMI < 30; obese, 30 ≤ BMI < 40; extremely obese, BMI ≥ 40. Years in the United States mean is among foreign born (*n* = 6,921).

**FIGURE 1:**
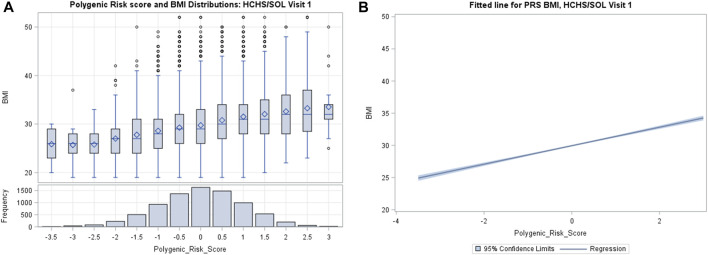
**(A)** Distribution of weighted polygenic risk scores for body mass index (PRS_BMI_) in participants from Hispanic Community Health Study/Study of Latinos (HCHS/SOL) visit 1 (2008–2011). **(B)** The fitted line is the regression of BMI and PRS_BMI_ adjusted for top five principal components, age, and sex.

PRS_BMI_ approximated a normal distribution in the population as shown in Figure 1A, while Supplementary Figure S3 shows that our BMI distribution was right skewed, which is consistent with previous population-based studies of countries like the United States (Ogden et al., 2007). We observed a monotonic gradient of overall increasing genetic risk and obesity as shown by the fitted line (Figure 1B). However, based on the tails of the distribution, we can visually determine that PRS_BMI_ is not strictly deterministic of obesity (Khera et al., 2019). For example, when looking at decile distribution, we find almost 2% of the 90th percentile or more of PRS_BMI_ score show phenotypic normal weight (18.5 > BMI > 25) category (Supplementary Figure S3). Conversely, below the 10th percentile, 2.3% of individuals were considered obese, and 0.1% were extremely obese. This distribution gradient of PRS_BMI_ persisted at the 6-years follow-up, visit 2. Consistent with previous work, women experienced elevated levels of BMI than did men at both visit 1 and visit 2 and herein show a trend in BMI (kg/m^2^) by PRS_BMI_, percentile for sex, which remains fairly consistent across visits.

Table 2 summarizes our evaluation of PRS performance by the variance explained (adjusted R^2^ of BMI and waist-to-hip ratio). We found that the PRS_BMI_ was well calibrated as evidenced by the 10.37% of variance explained in base model for BMI. Even though the full model choice was based on a continuous measure of BMI, the inferential model fit for waist-to-hip ratio performance was robust overall. However, the waist-to-hip measure is not a BMI-adjusted measure and should be interpreted as unadjusted. All outcome models fit, improved with the inclusion of interaction terms (age at immigration; healthy diet). Further evaluation of calibration, discrimination, and fit for outcome measures including dichotomous obesity to evaluate PRS utility can be found in the Supplemental Materials.

**TABLE 2:** Model fit for body mass index (BMI), waist-to-hip ratio, polygenic risk scores (PRSs), and their interactions with acculturation- and immigration-related variables in the up to 8,109 HCHS/SOL adults aged 20–76 years at visit 1 (2008–2011).

	Model adjustments	Adjusted r^2^					
		BMI			Waist-to-hip ratio		
**Model**		**Total**	**Males**	**Females**	**Total**	**Males**	**Females**
Base	BMI = PRS, 5 PCs, Study Center, Hispanic/Latino Background Group	0.1037	0.0916	0.1086	0.2289	0.2033	0.0896
	Base + Acculturation/Immigration	0.1187	0.1049	0.1275	0.2310	0.2071	0.0920
	Base + Acculturation/Immigration + Environmental	0.1612	0.1570	0.1718	0.2700	0.2483	0.1518
Full	Base + Acculturation/Immigration + Environmental + PRS × Age at Immigration + PRS × Healthy Diet	0.1656	0.1668	0.1749	0.2701	0.2515	0.1531

Note: All models account for HCHS/SOL complex survey design and sampling weights adjusted for top five PCs. Study center and Hispanic/Latino background group (Central American, Cuban, Dominican, Puerto Rican, South American, Mexican—ref). Factors that explained variability based on visit 1 backwards elimination inferential building were incrementally explored from base to full model, including *Acculturation/Immigration Measures*: Age at Immigration (Born in the United States, 0–5, 6–12, 13 **<** 20, arrived in the United States, 21 + years old—ref), Immigrant Generation (1st, 2nd—ref). Additional *Environmental Measures*: Education (<HS, ≥HS), Income (<30K USD, ≥30K USD), Employment Status (retired, not retired or employed, employed ≤ 35 h/week, employed > 35 h/week—ref), Diabetes Status (yes, no—ref); Prevalent Cardiovascular Disease (yes, no—ref); Sleep Duration (h/day); Healthy Diet JAMA (below 60th percentile by sex, above 60th percentile by sex—ref), Consumption of sweetened beverages (servings/day); Meets Physical Activity Guidelines (yes/no); Alcohol Use Level (no current use—ref, low-level use, high-level use); Cigarette Use (never—ref, current, former).

Next, we examined G×E among our initial model covariates (Supplementary Table S3). We find significant acculturation- and immigration-related interactions based on visit 1 BMI and PRS_BMI_ for age at immigration (US-born *p* < 0.1896, 0–5: *p* < 0.0022; 6–12: *p* < 0.728; 13–20: *p* < 0.2067) and the SASH Language Use subscale (*p* < 0.0357). There were other significant G×E interactions as well (e.g., healthy diet *p* < 0.0095; marital status, *p* < 0.0076; and sleep duration, *p* < 0.0219). We used these significant interactions to inform our model choice for a full model that included additional types of covariates and multiple interactions. We then leveraged visit 2 follow-up of the HCHS/SOL cohort to examine the stability of these interactions between the two time periods and observe similar effects reassessed 6 years after (Supplementary Table S3). We do not have dietary data at visit 2 and therefore could not examine the interaction between health diet and PRS-BMI longitudinally.

Our full model, which included significant predictors and interaction terms for visit 1, are presented in Tables 3, 4 (base model and fully adjusted model including PRS_BMI_ interaction terms). Consistent with our hypothesis that acculturation interactions would exacerbate the observed BMI (e.g., have a positive sign) and be significant, we observed significant interactions in both age at immigration and healthy diet (top 40th percentile). The validated subscale measures of SASH Social & Ethnic Relations and Language Use Subscales, as well as marital status, were not significant in our final multivariate model and therefore were excluded from our full model as well as those interaction terms.

**TABLE 3:** Polygenic risk score (PRS)—acculturation and environmental interactions for obesity (body mass index (BMI)) among HCHS/SOL participants for visit 1 (2008–2011) n = 8,109^a^, total and sex-stratified.

Model	Total				Males				Females			
	$\beta_{G}$ ^b^	SE	$\beta_{GxE}$	SE	$\beta_{G}$ ^b^	SE	$\beta_{GxE}$	SE	$\beta_{G}$ ^b^	SE	$\beta_{GxE}$	SE
**Base**	1.62***	0.09	-	-	1.44***	0.14	-	-	1.80***	0.12	-	-
**Full**	1.10***	0.13			0.79***	0.18			1.45***	0.17		
PRS_BMI_ ×												
Age at Immigration Born in the United States^c^			0.45	0.29			0.34	0.46			0.62	0.34
Age at Immigration 0–5^d^			1.12***	0.23			0.66	0.50			1.35*	0.60
Age at Immigration 6–12^e^			0.23	0.46			−0.04	0.37			0.38	0.73
Age at Immigration 13–20^f^			−0.10	0.22			−0.07	0.27			−0.10	0.31
JAMA Healthy Diet			0.46**	0.16			0.91***	0.24			−0.06	0.21

a Analytic sample includes participants with available genetic consent for study and complete case analysis.

b β_G_ for PRS_BMI_ 1-SD unit increase in PRS corresponds to 1-unit change in BMI (kg/m^2^).

c PRS_BMI_ × Age at Immigration (born in the United States compared with adult > 21 years old arrival).

d PRS_BMI_ × Age at Immigration (childhood 0–5 years old at first arrival compared with adult > 21 years old arrival).

e PRS_BMI_ × Age at Immigration (childhood 6–12 years old at first arrival compared with adult > 21 years old arrival).

f PRS_BMI_ × Age at Immigration (childhood 13–20 years old at first arrival compared with adult > 21 years old arrival).

*p < 0.05.

**p < 0.01.

***p < 0.001.

Note. All models account for HCHS/SOL complex survey design and sampling weights adjusted for top five PCs. Study center and Hispanic/Latino background group (Central American, Cuban, Dominican, Puerto Rican, South American, Mexican—ref). Full model additionally includes factors that explained variability based on visit 1 backwards elimination inferential building: *Acculturation/Immigration Measures*: Age at Immigration (Born in the United States, 0–5, 6–12, 13 **<** 20, arrived in the United States, 21 + years old—ref), Immigrant Generation (1st, 2nd—ref). Additional *Environmental Measures*: Age, Age^2^, Sex (female, male—ref); Education (<HS, ≥HS); Income (<30K USD, ≥30K USD); Employment Status (retired, not retired or employed, employed ≤ 35 h/week, employed > 35 h/week—ref); Diabetes Status (yes, no—ref); Prevalent Cardiovascular Disease (yes, no—ref); Sleep Duration (h/day); Healthy Diet JAMA (below 60th percentile by sex, above 60th percentile by sex—ref); Consumption of sweetened beverages (servings/day); Meets Physical Activity Guidelines (yes/no); Alcohol Use Level (no current use—ref, low-level use, high-level use); Cigarette Use (never—ref, current, former) significant interactions (PRS × Age at Immigration + PRS × JAMA Healthy Diet).

**TABLE 4:** Polygenic risk score (PRS)—acculturation and environmental interactions for obesity (waist-to-hip Ratio) among HCHS/SOL participants for visit 1 (2008–2011) n = 8,109^a^, total and sex-stratified.

Model	Total				Males				Females			
	$\beta_{G}$ ^b^	SE	$\beta_{GxE}$	SE	$\beta_{G}$ ^b^	SE	$\beta_{GxE}$	SE	$\beta_{G}$ ^b^	SE	$\beta_{GxE}$	SE
**Base**	0.007***	0.001	-	-	0.009***	0.001	-	-	0.004**	0.016	-	-
**Full**	0.005*	0.002			0.005*	0.002			0.003	0.002		
PRS_BMI_ ×												
Age at Immigration Born in the United States^c^			0.002	0.002			−0.002	0.004			0.005	0.004
Age at Immigration 0–5^d^			0.000	0.003			0.007	0.007			−0.004	0.010
Age at Immigration 6–12^e^			0.000	0.004			0.006	0.006			0.001	0.005
Age at Immigration 13–20^f^			−0.003	0.004			−0.002	0.003			−0.003	0.004
JAMA Healthy Diet			0.002	0.002			0.007**	0.003			−0.003	0.003

a Analytic sample includes participants with available genetic consent for study and complete case analysis.

b β_G_ for PRS_BMI_ 1-SD unit increase in PRS corresponds to 1-unit change in waist-to-hip ratio.

c PRS_BMI_ × Age at Immigration (born in the United States compared with adult > 21 years old arrival).

d PRS_BMI_ × Age at Immigration (childhood 0–5 years old at first arrival compared with adult > 21 years old arrival).

e PRS_BMI_ × Age at Immigration (childhood 6–12 years old at first arrival compared with adult > 21 years old arrival).

f PRS_BMI_ × Age at Immigration (childhood 13–20 years old at first arrival compared with adult > 21 years old arrival).

*p < 0.05.

**p < 0.01.

***p < 0.001.

Note: All models account for HCHS/SOL complex survey design and sampling weights adjusted for top five PCs. Study center and Hispanic/Latino background group (Central American, Cuban, Dominican, Puerto Rican, South American, Mexican—ref). Full model additionally includes factors that explained variability based on visit 1 backwards elimination inferential building: *Acculturation/Immigration Measures*: Age at Immigration (born in the United States, 0–5, 6–12, 13 **<** 20, arrived in the United States, 21 + years old—ref), Immigrant Generation (1st, 2nd—ref). Additional *Environmental Measures*: Education (<HS, ≥HS), Income (<30K USD, ≥30K USD), Employment Status (retired, not retired or employed, employed ≤ 35 h/week, employed > 35 h/week—ref), Diabetes Status (yes, no—ref); Prevalent Cardiovascular Disease (yes, no—ref); Sleep Duration (h/day); Healthy Diet JAMA (below 60th percentile by sex, above 60th percentile by sex—ref), Consumption of sweetened beverages (servings/day); Meets Physical Activity Guidelines (yes/no); Alcohol Use Level (no current use—ref, low-level use, high-level use); Cigarette Use (never—ref, current, former) significant interactions (PRS × Age at Immigration + PRS × JAMA Healthy Diet).

**FIGURE 2:**
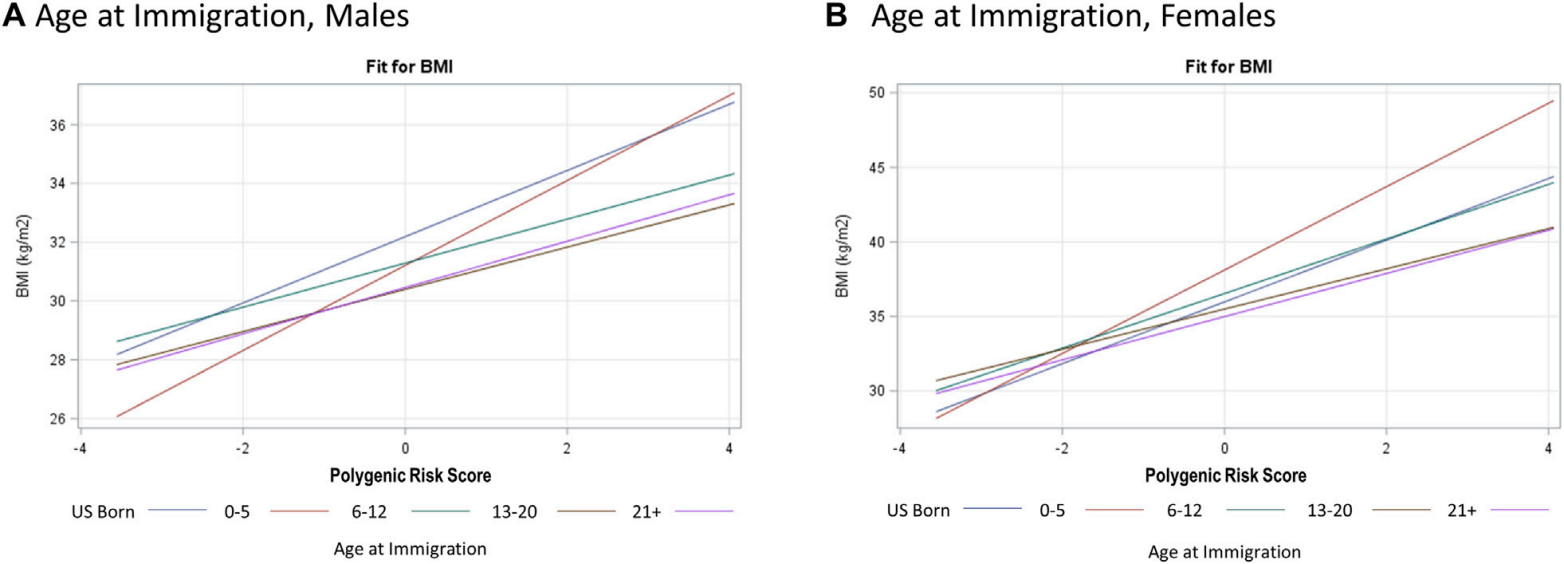
Hispanic Community Health Study (HCHS) visit 1 (2008–2011) sex-stratified effect plots showing the gene–environment interaction for PRS_BMI_ × Age at Immigration **(A, B)** from fully adjusted model. The crossing lines represent interaction and shows that the effect of overall genetic risk on body mass index (BMI) is different for different values of age at immigration.

**FIGURE 3:**
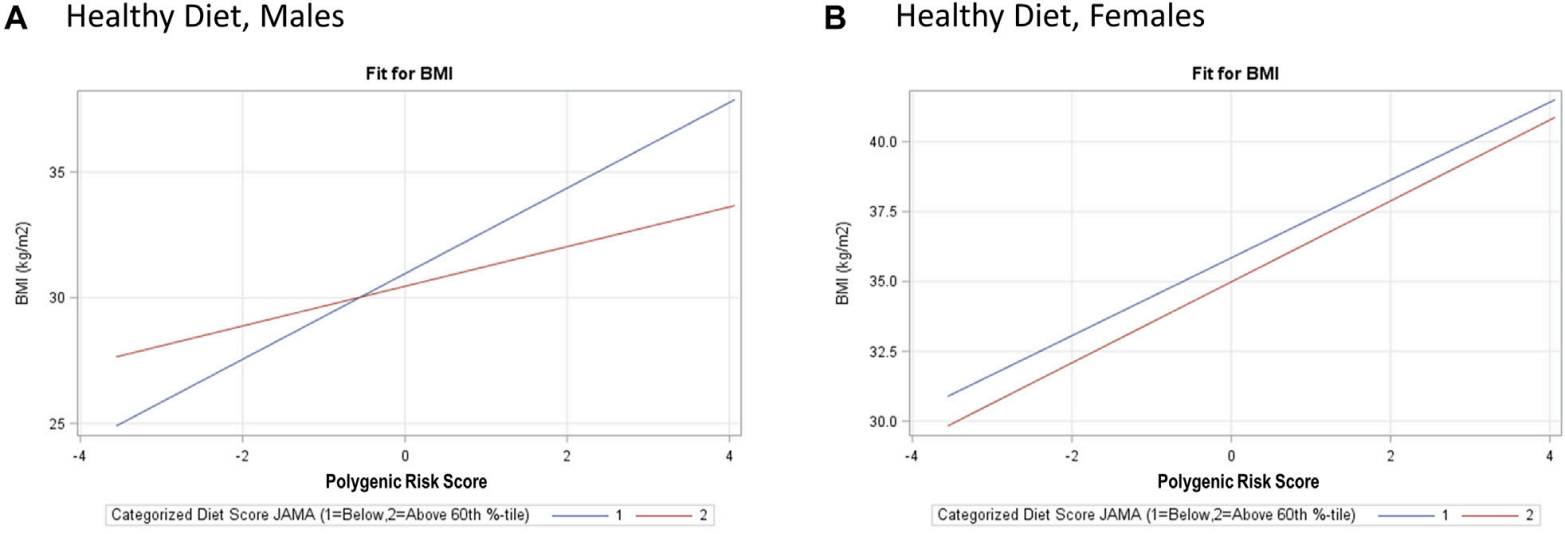
Hispanic Community Health Study (HCHS) visit 1 (2008–2011) sex-stratified effect plots showing the gene–environment interaction for and PRS_BMI_ × Healthy Diet **(A, B)** from the fully adjusted model. The crossing lines represent interaction and show the effect of overall genetic risk on body mass index (BMI) is different for different values of healthy diet for males, with a notable lack of interaction for PRS_BMI_ and healthy diet for females.

Based on our full model, a 1-SD increase in PRS_BMI_ corresponds with a 1.10 kg/m^2^ increase in BMI after adjusting for top five PCs of ancestry, study center, and Hispanic/Latino background group as well as acculturation and other environmental variables that explained a 6% increase in the variation explained in our model (Table 3, BMI). Due to the presence of interaction terms, we can interpret this PRS_BMI_ estimate as conditional on the different values of age at immigration (compared with adult arrival) and JAMA healthy diet (bottom 60th percentile diet for sex compared with top 40th percentile). This elevated risk of BMI by 1-SD increase in PRS_BMI_ increase and with a larger PRS_BMI_-related increase in BMI for women 1.45 kg/m^2^ compared with men 0.79 kg/m^2^ in sex-stratified analysis (full model). Figure 2 illustrates that at low values of PRS_BMI_, for example, we observe that adult arrival age at immigration group, for both males and females, has a higher BMI. However, at higher values of PRS_BMI_, adult arrival age at immigration has a lower BMI, effectively crossing over. Most notably, the interaction terms in the stratified models are only significant for men and only for diet, suggesting that these differences may further drive the significance in the full total model. These effect plots show interaction effects (Figure 3) by PRS_BMI_ on BMI wherein the effect of the G×E value on BMI depends on PRS_BMI_, with the exception of healthy diet in females, which has parallel lines and no significant interaction (*p* = 0.7938).

Finally, we conducted exploratory analyses examining the fully adjusted interaction model stratified by genetic ancestry background Hispanic/Latino group (Table 5). We compare the difference in each model with and without the PRS_BMI_ main effect to evaluate the incremental difference. In models without the PRS_BMI_, there was more variation by genetic background group. However, adjusted r^2^ and effect improves with the inclusion of the PRS_BMI_ both across background groups and in proportion of the variance explained. The PRS_BMI_ estimates were comparable across the background Hispanic/Latino groups, while the PRS interaction terms had more variability by background. In particular, the South American background group had a larger standard error and beta estimate for PRS age at immigration US-born and age 0–5 compared with adult age at immigration, which is discordant with the estimates for other groups.

**TABLE 5:** Exploratory analysis of incremental validity of polygenic risk score (PRS)—acculturation and sociocultural interactions for obesity (BMI) among HCHS/SOL participants for visit 1 (2008–2011) n = 8,109, stratified by background Hispanic/Latino group.

	Central American		Cuban		Dominican		Puerto Rican		South American		Mexican	
n	n = 897		n = 1,429		n = 728		n = 1,431		n = 576		n = 3,048	
Weighted n	n = 638.07		n = 1,847.3		n = 711.30		n = 1,329.9		n = 442.03		n = 2,823.3	
Model 1												
r^2^	0.0581		0.0351		0.0374		0.0681		0.0608		0.0149	
+ PRS r^2^	0.1283		0.1381		0.1189		0.1475		0.1388		0.0789	
Difference	0.0702		0.1030		0.0815		0.0794		0.0780		0.0640	
Model 2												
r^2^	0.0718		0.0555		0.0658		0.0872		0.0745		0.0394	
+ PRS r^2^	0.1430		0.1580		0.1431		0.1632		0.1480		0.1017	
Difference	0.0712		0.1026		0.0773		0.0760		0.0735		0.0623	
Model 3												
r^2^	0.1221		0.1274		0.1431		0.1651		0.1573		0.1066	
+ PRS r^2^	0.1826		0.2165		0.2032		0.2269		0.2226		0.1576	
Difference	0.0605		0.0891		0.0601		0.0618		0.0653		0.051	
Model 4												
r^2^	0.1876		0.2218		0.2317		0.2309		0.2444		0.1664	
+ PRS r^2^												
Difference												
**Model main effect**	**β**	**SE**	**β**	**SE**	**β**	**SE**	**β**	**SE**	**β**	**SE**	**β**	**SE**
1: PRS_BMI_	1.54***	0.22	1.69***	0.13	1.75***	0.28	1.88*	0.22	1.55***	0.28	1.46***	0.18
2: PRS_BMI_	1.55***	0.22	1.69***	0.13	1.71***	0.27	1.85***	0.21	1.52***	0.27	1.45***	0.18
3: PRS_BMI_	1.48***	0.22	1.60***	0.12	1.54***	0.24	1.69***	0.22	1.46***	0.23	1.35***	0.16
4: PRS_BMI_	1.37***	0.28	1.35***	0.20	0.55	0.46	0.98*	0.42	1.73***	0.31	0.91***	0.21
4: Interactions												
PRS_BMI_ ×												
Born in the United States^c^	0.55	0.77	0.04	0.65	1.58	1.11	0.08	0.45	−1.74*	0.86	1.09*	0.49
0–5 years	0.16	0.69	2.27**	0.80	−0.57	1.0	1.13	0.70	−4.71**	1.77	1.59*	0.71
6–12 years	−1.47	1.14	−0.59	0.79	4.05	2.44	0.54	0.57	2.08*	0.95	0.20	0.54
13–20 years	−0.62	0.81	0.49	0.48	−0.61	0.48	0.34	0.55	0.19	1.00	0.07	0.39
Healthy Diet	0.41	0.45	0.31	0.27	1.08*	0.52	0.59	0.40	−0.35	0.55	0.28	0.39

*p < 0.05.

**p < 0.01.

***p < 0.001.

Note: Nested incremental modeling as follows.

Model 1: BMI = PRS, 5 PCs, age, age^2^, sex, Hispanic/Latino background group, study center.

Model 2: BMI = PRS, 5 PCs, age, age^2^, sex, Hispanic/Latino background group, study center + Acculturation/Immigration.

Model 3: BMI = PRS, 5 PCs, age, age^2^, sex, Hispanic/Latino background group, study center + Acculturation/Immigration + Environmental.

Model 4: BMI = PRS, 5 PCs, age, age^2^, sex, Hispanic/Latino background group, study center + Acculturation/Immigration + Environmental + PRS × Age at Immigration + PRS × JAMA Healthy Diet.

## Discussion

Among this ancestrally and socioculturally diverse sample of Hispanic/Latinos, we identify similar G×E interactions reported in other studies (Qi et al., 2014; Nakamura et al., 2016; Reddon et al., 2016; Rask-Andersen et al., 2017) and novel G×E interactions for age at immigration and healthy diet (full model). In other words, we observe that the PRS_BMI_ effect on BMI was different for different values of acculturation and environmental factors alike. Overall, we found that an increase in polygenic risk is strongly associated with an increase in BMI (Khera et al., 2019), and despite the estimated PRS_BMI_, coefficients were moderately sensitive to model specification across all models and were directionally consistent, and statistically significance remained similar. When we modeled this association using visit 2 BMI data, we observed less of a genetic effect for elevated BMI. These models highlight that PRS_BMI_ was not exclusively deterministic of BMI either cross-sectionally or longitudinally, and the etiology of obesity remains highly multifactorial in nature.

We identified differential sex-stratified effects by examining the same models among men and women separately. Herein, we find that dietary quality and acculturation interaction display sex-specific patterns with age at immigration significant for females only and diet significant for males only. This is consistent with previous PRS literature regarding sex differences in cancer outcomes (Fritsche et al., 2018; Meisner et al., 2020; Roberts et al., 2020) but has not been widely reported in the growing literature on PRSs for obesity (Khera et al., 2019; Torkamani and Topol 2019; Lee et al., 2021). Importantly, our study only captured the binary biological sex categories of male and female, so we are unable to contextualize these differences by broader gender-identity and gender-related processes, which we hypothesize may be underlying some of these observed differences. For example, we do not know to what degree the interaction of dietary score with genetic risk is because males have a different dietary pattern (Wardle et al., 2002) versus biological sex as a genetic modifier of nutrigenomic pathways.

In our study, we examined dietary interaction, which may represent both direct (immigration) and potentially indirect mechanisms of acculturation. We were able to examine some dietary measures that provide additional context, such as consumption of sugar-sweetened beverages, in which males on average consume 2.2 servings per day compared with 1.3 for females. This may help explain our findings that dietary patterns modify genetic risk of elevated BMI for men. In sensitivity analysis, we used an alternate dietary variable, the AHEI, modeled continuously and dichotomized similarly to the original variable for comparison. However, this alternative variable is not significant in the full model. Both dietary measures used National Cancer Institute (NCI) method for predicting usual intake based on 24-h recall, but they measured different dietary components. Recent work highlighting a significant G×E interaction for dietary fiber (Hüls et al., 2021) with polygenic risk for obesity among pan-European children may help explain these differences, as fiber is one of the four components of the Healthy Diet JAMA top 40th percentile variable. Additionally, the AHEI includes two variables already in the model, sugar-sweetened beverages and alcohol use, so the effect may be attenuated for this specific dietary measure compared with the original measure. Nonetheless, it would be important to confirm additional dietary interactions particularly for sex-specific models in future analyses.

In this work, we sought to elucidate the role of acculturation, immigration, sociodemographic lifestyle, and other environmental factors on genetic risk for elevated BMI. In our inferential model building, we ultimately excluded some of the measures that we had conceptually hypothesized would likely explain a portion variability of PRS_BMI_ on BMI or perhaps serve as a better (validated) marker of acculturation or acculturative stress (e.g., the SASH Language Use, Social & Ethnic Relations Validated (Marin et al., 1987) Subscales, or Ethnic Identification score). This may be due in part to the fact that proxy measures of acculturation and immigration were stronger predictors of the same inter-related process and thus retained in our model building. For example, we used age at immigration, as it conceptually informed our model and was significant, and immigration measures represent a combination of broad acculturation trends and information on timing of first exposure to the United States. Yet such proxy measures may be less helpful at explaining life course changes or lifestyle interventions, as they remain fixed exposure, whereas other acculturation measures such as SASH Language Use subscale can be assessed in a more dynamic manner. In subsequent sensitivity analyses, we examined the effect on PRS_BMI_ when we recapture or include alternate measures of acculturation (SASH-Language) that were otherwise not significant in our final modeling. We found directionally consistent results (i.e., smaller PRS coefficient with inclusion of interaction) but less significant interaction terms for these measures. Although conceptually we would expect that as time in the United States increased more variability would be captured by measures of increased acculturation, we did not measure this in our current study.

We reported age at immigration as a significant interaction term included in our full model. This measure aimed to approximate the interplay of the acculturative process as both duration and timing of immigration to the United States. Previous work reported significance differences in obesity outcomes by duration of exposure comparing migration before age 20 to later age at migration in adulthood (Roshania et al., 2008). To test our hypothesis that these differences may be attributable to *in utero* and early life exposures, we further categorized the less than 20 years, or early, age at migration as 0–5, 6–12, and 13–20 years old and found the age at immigration 0–5 group compared with age at immigration 21 or older had a significant interaction with PRS_BMI_. Importantly, compared with Roshania et al., where they examined recent immigrants (5 years mean duration of US residence), our target population sample resided in the United States longer (average 20.32 years). While our findings may also represent *in utero* or early life exposure mechanisms, we cannot conclusively detangle them from duration of exposure. There may also be confounding by the selection into migration (Murphy et al., 2017). Further work is needed to elucidate the sensitivity of this mechanism to critical periods of development, such as early childhood and obesity risk in migrant groups. A challenge in considering interpretation of PRS_BMI_ is the role of lifestyle and other environmental factors. This wide array of interaction also shows that there is potential modification of the genetic risk modeled by PRS_BMI_ by many different factors and highlight the importance of these environmental conditions for optimizing PRS_BMI_ utility. Recent work has used electronic health records to identify risk factors for polygenic risk and prediction of prevalent disease status (Dashti et al., 2019). This type of integration of individual biologic data with the social determinants of health information as part of electronic health records is growing in practice could greatly inform what are the future clinical applications of PRSs (Gottlieb et al., 2013; Cantor and Thorpe 2018; Chen et al., 2020). Any consideration of risk for obesity should also consider environmental components that contribute to that risk. This study may help inform the characterization and advancement towards mature polygenic and environmental risk scores for obesity with clinical applications.

Our study is not without limitations. We could potentially have overfitting in our model (Mak et al., 2018) due to any overlap between the discovery data and the shrinkage applied to the GWAS effect size based on HCHS/SOL data, but it is unlikely given the different data sources. The study design involves complex sampling, and therefore, we utilized complex survey methodology in our analysis. However, the framework for model evaluation and building are less well established compared with non-survey methods and may have resulted in the introduction of additional Type 1 error (Hahs-Vaughn et al., 2011). We did employ a domain analysis that extended the standard errors to include the study target population (*n* = 16,415) in our estimations. Additionally, the purpose of this analysis was not strictly risk prediction but to illustrate the ability of polygenic risk prediction at informing G×E interaction modeling.

In order to assess the genetic information, we restricted our sample to only those who consented to share genetic data and both visit 1 and reconsented at visit 2. We limited our analysis to complete cases and may therefore have lost some of the representativeness of our data or are missing not at random; however, all standard error estimates presented are calculated, adjusting for the full target population using complex survey methodology. In this diverse sample of Hispanic/Latinos, we found a PRS_BMI_, derived from a large European ancestry GWAS explained a considerable portion of variability in BMI, with only subtle differences in distributions by Hispanic/Latino ancestry groupings (Table 5). The discovery data (Yengo et al., 2018) and target data (HCHS/SOL) were derived from different populations, indicating that there may be some degree of transportability of PRS_BMI_ for BMI across these two populations. However, we cannot rule out that some of the observed interactions may be due to having an imperfect estimation of genetic effect relation to a weaker instrument based on European-weighted ancestry. While the predominantly European ancestry effect sizes were used as weights in the PRS calculation used herein, other weights were explored as part of our preliminary work (not reported). For example, we leveraged Hispanic/Latino effect sizes provided by the investigators of the Hispanic/Latino Anthropometry Consortium (Young et al., 2018), but these yielded PRS that explained less variance in BMI than when European-ancestry weights were used. We cannot rule out that the PRS measure is more sensitive to sample size compared with ancestry, as the difference is order of magnitude for the European-derived weights (Yengo et al., 2018). This highlights the difficulty of parsing a multifactorial etiology (e.g., genetic, environmental, and G×E) when the best practices for estimating trans-ancestral or sub-population-specific PRS—especially in light of their smaller available sample size—are still being developed.

Our model fit performed well for a model examining a complex disease such as obesity. Notably, we found the combination of predictors of genetic risk and environmental interactions to include two statistically significant interactions with PRS_BMI_. Although these findings are exploratory, taken together, they warrant future research and endeavors designed to better understand and model risk between the genetic architecture of obesity and environmental factors broadly speaking. Furthermore, future studies should consider ways to model gender and sex differences, as well as adopt a longitudinal approach to better account for age-related BMI changes. Our preliminary observation of G×E interactions across a 6-years time period implies that PRS_BMI_ and environmental factors (either static or time-varying) should be jointly considered when considering the obesity prevention or counseling effects within the realm of precision medicine.

## Data Availability

The data presented in the Hispanic Community Health Study/Study of Latinos (HCHS/SOL) are deposited in dbGaP repository, accession number, phs000810.v1.p1. Additionally, the Hispanic Community Health Study/Study of Latinos (HCHS/SOL) data used in this article are publicly available following an approved article proposal. The investigators’ website can be found here: http://www.cscc.unc.edu/hchs/.

## Supplementary Material

Supplementary Figure 1 Study Inclusion/Exclusion Flow Chart

Supplementary Figure 2 Three-Stage Inferential Model Building Diagram.
