# Supplementary figures and images for: Findings from the Hispanic Community Health Study/Study of Latinos on the Importance of Sociocultural Environmental Interactors: Polygenic Risk Score-by-Immigration and Dietary Interactions
Source: Front Genet. 2021 Dec 6;12:720750. doi: 10.3389/fgene.2021.720750 (PMC8685455; doi:10.3389/fgene.2021.720750)

## Supplemental Figure 1.

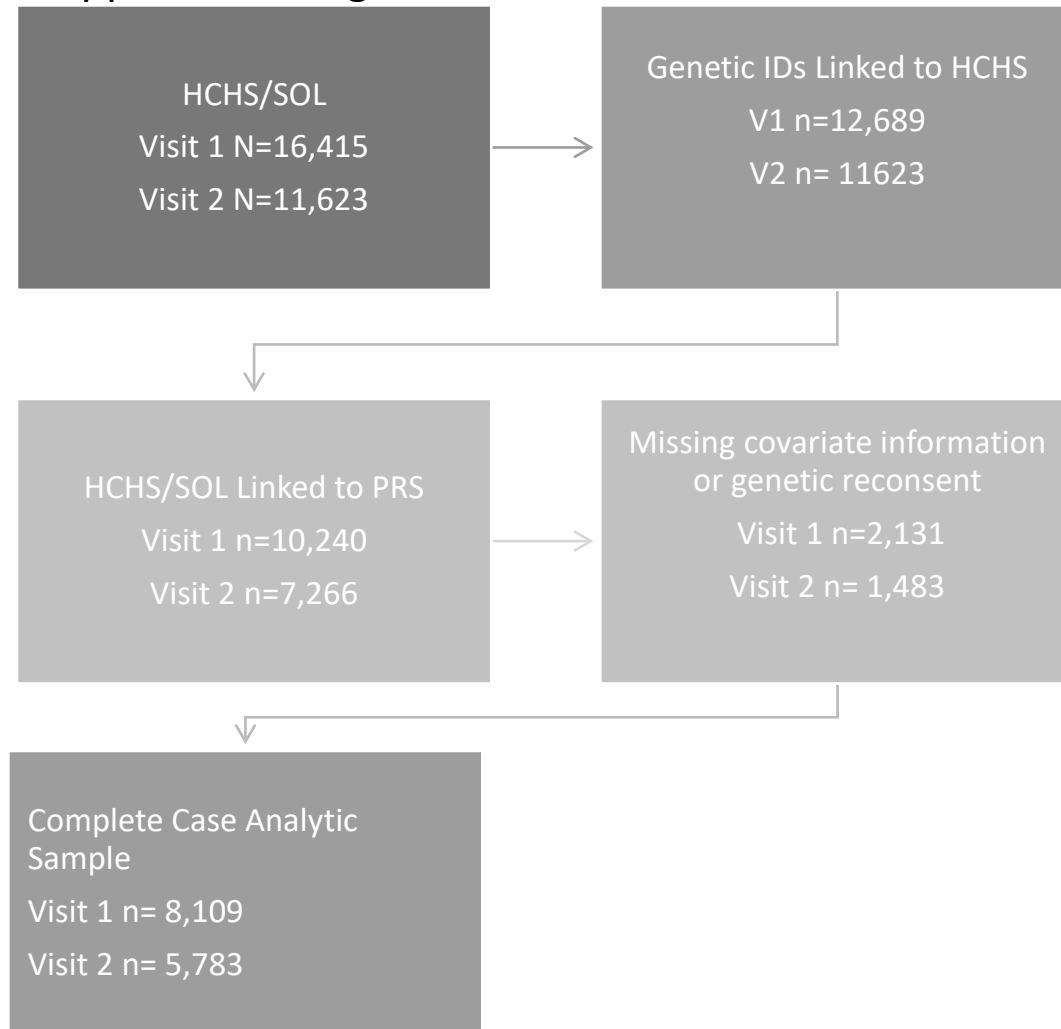

Supplemental Figure 2.

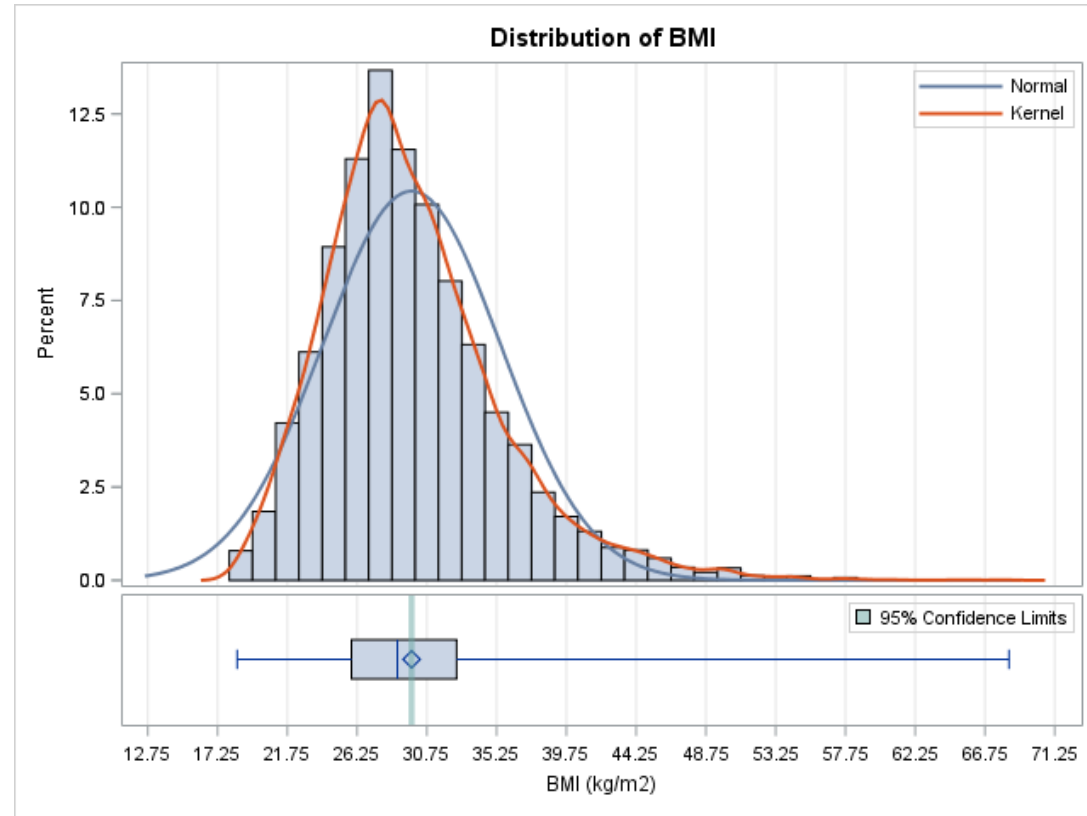

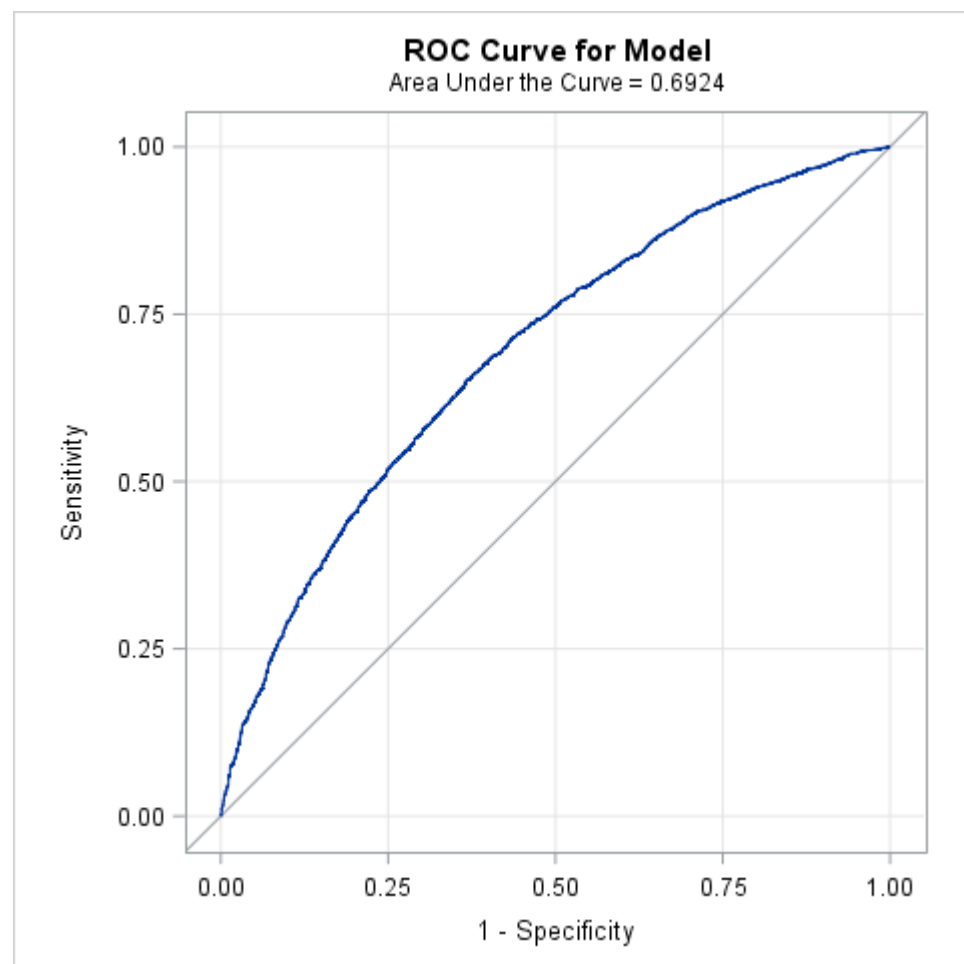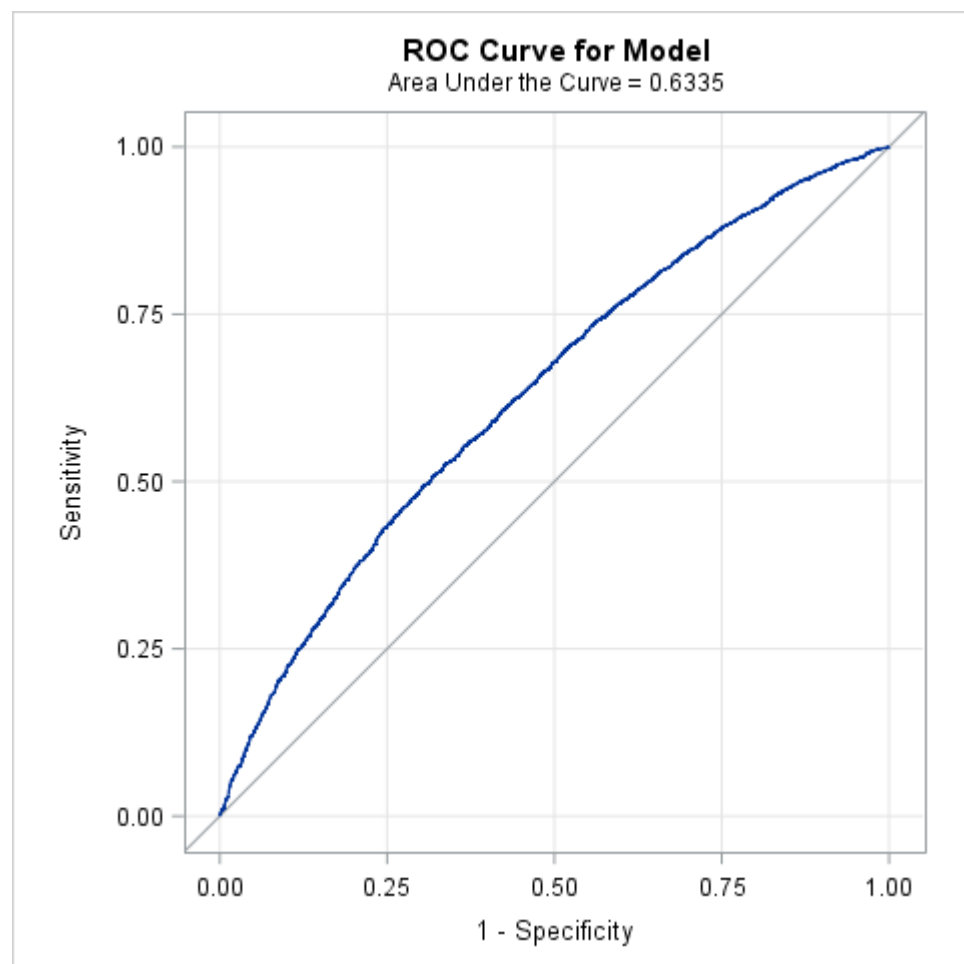

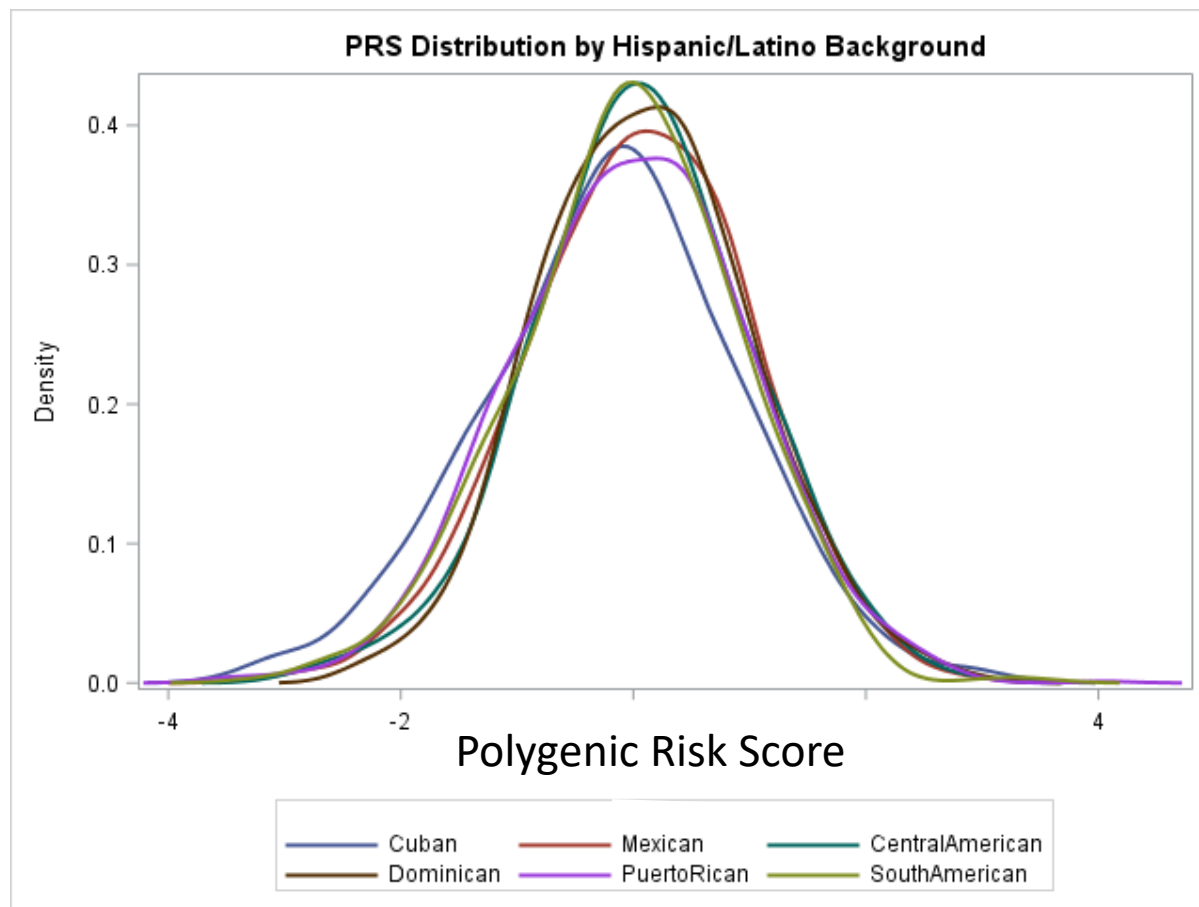

### Genetic Background BMI Mean Differences

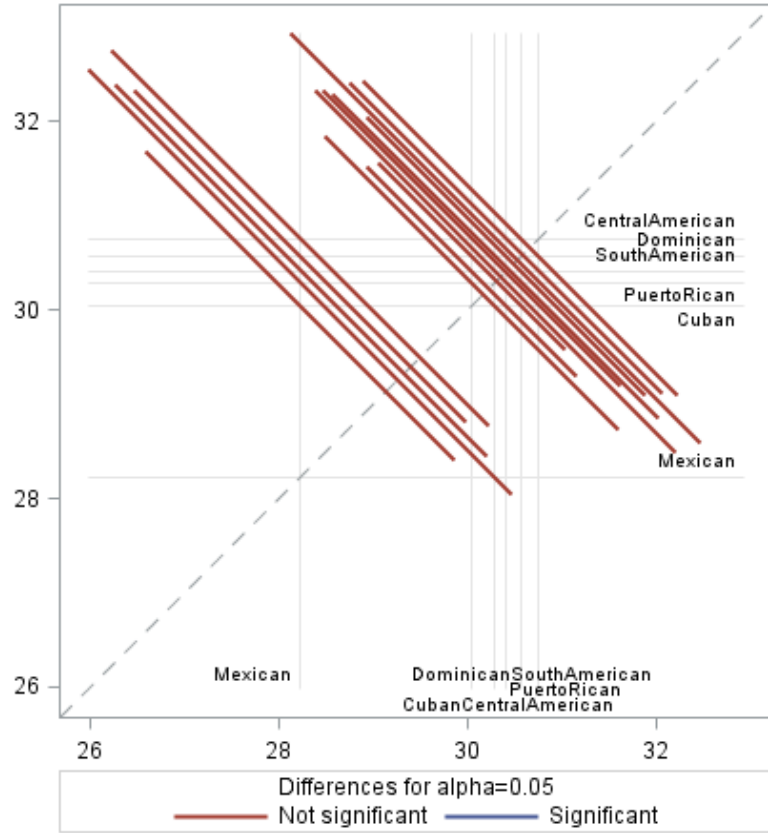

Supplement: Supplementary file 1 [file DataSheet1.PDF]
