# Supplementary material for: Findings from the Hispanic Community Health Study/Study of Latinos on the Importance of Sociocultural Environmental Interactors: Polygenic Risk Score-by-Immigration and Dietary Interactions
Source: Front Genet. 2021 Dec 6;12:720750. doi: 10.3389/fgene.2021.720750 (PMC8685455; doi:10.3389/fgene.2021.720750)
Supplement: Supplementary file 2 [file DataSheet1.docx]

Supplemental Materials

| **Supplemental Table 1.**  Three-stage model choice informed by augmented backwards elimination (BE) and interaction significance evaluation. | | | | | | | | | | | | | | |
| --- | --- | --- | --- | --- | --- | --- | --- | --- | --- | --- | --- | --- | --- | --- |
| **VARIABLE INFORMATION** | | | | | | | | | | | | | | |
| Explanatory passive variables | | PRS and top 5 principal components, study center, Hispanic/Latino background | | | | | | | | | | | | |
|  | |  | | | | |  | | | | | | | |
| Sociocultural Environment: Acculturation and Immigration measures | | Survey Mean Centered SASH Language Subscale, Survey mean Centered SASH Socio and Ethnic Relations Subscale, Age at Immigration (US Born, 0-5, 6-12, 13-20, 21+ -ref), Immigrant Generation (1^st^, 2^nd^-ref), Cohort of Birth Year (Pre-1980, Post-1980), | | | | | | | | | | | | |
|  | |  | | | | |  | | | | | | | |
| Additional Environment measures | | sex, age, age^2^, marital status (married and living with partner or not), Education (<HS, >HS), Income (<30K USD, >30K USD), Employment status (Retired, Not Retired or Employed, Employed <35 hrs/wk, Employed >35 hrs/wk-ref), diabetes status (yes, no); prevalent cardiovascular disease (yes, no); sleep duration (hours per day); consumption of sweetened beverages (servings/day); physical activity guidelines met (yes/no); alcohol use level (no current use, low level use, high level use); cigarette use (never, current, former); CES-D Depression 10 item Summary Score, Ethnic Identity Score, Healthy Diet JAMA (Below 60^th^ percentile, Above 60^th^ percentile –ref) | | | | | | | | | | | | |
| Survey mean centered variables (continuous) | | Sleep duration (**7.884428)**, sugar sweetened beverages (**1.780906**), CEDS-10 summary scale (**6.963373)**, ethnic Identity Score (**3.183784)**, Years lived in US (**17.876142**), SASH Social and Ethnic Relations (**2.228099)** , SASH Language Use (**2.040466** ), Alternative Healthy Eating Index 2010 (**48.162688**) | | | | | | | | | | | | |
|  | |  | | | | |  | | | | | | | |
| Backwards Elimination Global model for Stage 1 | | BMI= intercept +PRS+ 5 PCs (ev1, ev2, ev3, ev4, ev5) + Study Center (Bronx, Chicago, San Diego, Miami-ref) + Hispanic/Latino background (Cuban, Dominican, Puerto Rican, Central American, South American, Mexico-ref) + Sociocultural Environmental variables + Additional Environmental measures + error | | | | | | | | | | | | |
| **STAGE 1** | | | | | | | | | | | | | | |
| Stage 1: Model building using passive variables, sociocultural environmental variables, sequentially excluded from the model based on BE alpha=0.2, or change in criterion tau 0.05 | | | | | | | | | | | | | | |
|  |  | | | | | |  | |  | | | | | |
| Covariate | Pr>\|t\| | | **F Value** | | | Pr>F | Backwards Elimination Exclusion Criteria | | | | | | | |
| Cohort 1980 | 0.9016 | | 0.02 | | | 0.9016 | Omit variable by alpha=0.2 | | | | | | | |
|  | | | | | | |  | | | | | | | |
| SASH Social and Ethnic Relations | 0.5087 | | 0.44 | | | 0.5087 | Omit variable by alpha=0.2 | | | | | | | |
|  | | | | | | |  | | | | | | | |
| SASH Language Use | 0.5371 | | 0.38 | | | 0.5371 | Omit variable by alpha=0.2 | | | | | | | |
|  | | | | | | |  | | | | | | | |
| Ethnic Identity Score | 0.4408 | | 0.59 | | | 0.4408 | Omit variable by alpha=0.2 | | | | | | | |
|  | | | | | | |  | | | | | | | |
| CESD-10 Depression Scale | 0.4204 | | 0.65 | | | 0.4204 | Omit variable by alpha=0.2 | | | | | | | |
|  | | | | | | |  | | | | | | | |
| Immigrant Generation | 0.3743 | | 0.79 | | | 0.3743 | Immigrant Generation p-value 0.3743 > 0.20, however large change in criterion Passive variable ev4 (Principal Component variable): stand. delta= \| -2.208991\| >= 0.05;  *Variable is a confounder and will be kept | | | | | | | |
|  | | | | | | |  | | | | | | | |
| Income | 0.3031 | | 1.06 | | | 0.3031 | INCOME p-value 0.3493 > 0.20, however large change in criterion Passive variable ev3: stand. delta= \| 1.996667\| >= 0.05;  *Variable is a confounder and will be kept | | | | | | | |
|  | | | | | | |  | | | | | | | |
| Survey Mean Centered Sugar Sweetened Beverages | 0.2619 | | 1.26 | | | 0.2619 | Survey Mean Centered Sugar Sweetened Beverages p-value 0.2643 > 0.20 , however large change in criterion passive variable ev2: stand. delta= \| 0.732799\| >= 0.05; *Variable is a confounder and will be kept; | | | | | | | |
|  | | | | | | |  | | | | | | | |
| Education | 0.2756 | | 1.19 | | | 0.2756 | *Remove EDUCATION_C2 p-value 0.2680 < p0.20, however large change in criterion passive variable ev2: stand. delta= \| -1.665616 \| >= 0.05;  *Variable is a confounder and will be kept; | | | | | | | |
|  | | | | | | |  | | | | | | | |
| Marital | 0.8313 | | 0.05 | | | 0.8313 | Omit variable by alpha=0.2 | | | | | | | |
|  |  | | | | | | | | | | | | | |
| *All passive explanatory variables or active confounders included in full model: R-Square 0.1645 , Adjusted R-Square 0.1612 | | | | | | | | | | | | | | |
| Full Model | | | |  | | | | | | | | | | |
| Covariates *Excluded* | | | | Cohort 1980 ; Sash Language ;Sash Social and Ethnic Relations; Immigrant Generation Ethnic Identity ; CESD-10 Depression Scale; marital | | | | | | | | | | |
| Covariate *Include*d | | | | Backward Elimination Modeling | | | | | | **F Value** | | Pr>F | | |
|  | | | | PRS | | | | | | 320.21 | | <.0001 | | |
|  | | | | DIABETES | | | | | | 83.09 | | <.0001 | | |
|  | | | | Age^2^ | | | | | | 47.54 | | <.0001 | | |
|  | | | | Age | | | | | | 43.54 | | <.0001 | | |
|  | | | | Sex | | | | | | 33.33 | | <.0001 | | |
|  | | | | Sleep Duration | | | | | | 27.00 | | <.0001 | | |
|  | | | | Cigarette Use | | | | | | 17.84 | | <.0001 | | |
|  | | | | Physical Activity | | | | | | 11.03 | | 0.0009 | | |
|  | | | | Cardiovascular Disease | | | | | | 9.95 | | 0.0017 | | |
|  | | | | JAMA Healthy Diet | | | | | | 9.05 | | 0.0027 | | |
|  | | | | Age at Immigration | | | | | | 5.61 | | 0.0002 | | |
|  | | | | Employment Status | | | | | | 4.84 | | 0.0025 | | |
|  | | | | Alcohol Use Level | | | | | | 2.63 | | 0.0727 | | |
|  | | | | Sugar Sweetened Bev | | | | | | 1.26 | | 0.2619 | | |
|  | | | | Education >HS | | | | | | 1.19 | | 0.2756 | | |
|  | | | | Income >30K | | | | | | 1.06 | | 0.3031 | | |
|  | | | | Immigrant Generation | | | | | | 0.79 | | 0.3743 | | |
|  | | | |  | | | | | |  | |  | | |
| **STAGE 2** | | | | | | | | | | | | | |  |
| Stage 2: (1) Evaluate interaction terms for significance to add to the full model using significant interactions (p<0.05) from independent GxE modeling^a^ (see Supplemental Table 3 for additional information). | | | | | | | | | | | | | |  |
|  | | | | | | | | | | | | | |  |
| **^a^** Model =Passive Variables + Environmental + PRS*Environmental variables from STAGE, model results in Supplemental Table 3 | | | | | | | | | | | | | |  |
| **Environmental Interactions with PRS^a^** | | | | | Main Pr>\|t\| | | | GxE Pr>\|t\| | | | F value | | Pr>F |  |
| PRS*Sex | | | | | <.0001 | | | 0.0355 | | | 4.44 | | 0.0355 |  |
| PRS*SASH Language | | | | | 0.0514 | | | 0.0357 | | | 4.43 | | 0.0357 |  |
| PRS*Age at Immigration | | | | | 0.001 | | | 0.1896 | | | 3.39 | | 0.0093 |  |
|  | | | | | 0.0043 | | | 0.0022 | | |  | |  |  |
|  | | | | | 0.728 | | | 0.728 | | |  | |  |  |
|  | | | | | 0.2067 | | | 0.2067 | | |  | |  |  |
| PRS*Marital | | | | | 0.631 | | | 0.0076 | | | 7.18 | | 0.0076 |  |
| PRS*JAMA Healthy Diet | | | | | 0.004 | | | 0.0095 | | | 6.76 | | 0.0095 |  |
| PRS*Sleep Duration | | | | | <.0001 | | | 0.0219 | | | 5.28 | | 0.0219 |  |
| **Significant (p<0.05): Age at Immigration, JAMA Healthy Diet, Survey Mean-Centered Sleep Duration, Marital, SASH Language, Sex** | | | | | | | | | | | | | |  |
| Stage 2: (2) Evaluate the 6 significant main interactions assessed jointly, until only significant interactions remain using a correction for multiple comparisons | | | | | | | | | | | | | |  |
| **Interaction effects assessed jointly** | | | | | | | | **GxE Pr>\|t\|** | | | **F value** | | **Pr>F** |  |
| **Joint Model 1:** | | | | |  | | |  | | |  | |  |  |
| PRS*Sex | | | | |  | | | 0.0441 | | | 4.07 | | 0.0441 |  |
| PRS*SASH Language ++ | | | | |  | | | 0.4742 | | | 0.51 | | 0.4742 |  |
| PRS*Age at Immigration | | | | | US Born | | | 0.5830 | | | 1.15 | | 0.3301 |  |
|  | | | | | 0 < 5 | | | 0.0783 | | |  | |  |  |
|  | | | | | 6<12 | | | 0.9608 | | |  | |  |  |
|  | | | | | 13 < 21 | | | 0.5079 | | |  | |  |  |
| PRS*Marital | | | | |  | | | 0.1190 | | | 2.44 | | 0.1190 |  |
| PRS*JAMA Healthy Diet | | | | |  | | | 0.0102 | | |  | |  |  |
| PRS*Sleep Duration | | | | |  | | | 0.0320 | | | 4.62 | | 0.0320 |  |
|  | | | | |  | | |  | | |  | |  |  |
| **Joint Model 2:** | | | | |  | | |  | | |  | |  |  |
| PRS*Sex | | | | |  | | |  | | | 3.89 | | 0.0489 |  |
| PRS*Age at Immigration | | | | | US Born | | | 0.1462 | | | 2.11 | | 0.0778 |  |
|  | | | | | 0 < 5 | | | 0.0133 | | |  | |  |  |
|  | | | | | 6<12 | | | 0.7222 | | |  | |  |  |
|  | | | | | 13 < 21 | | | 0.6229 | | |  | |  |  |
| PRS*Marital++ | | | | |  | | | 0.1143 | | | 2.50 | | 0.1143 |  |
| PRS*JAMA Healthy Diet | | | | |  | | | 0.0112 | | | 6.48 | | 0.0112 |  |
| PRS*Sleep Duration | | | | |  | | | 0.0308 | | | 4.69 | | 0.0308 |  |
|  | | | | |  | | |  | | |  | |  |  |
| **Joint Model 3:** | | | | |  | | |  | | |  | |  |  |
| PRS*Sex | | | | |  | | | 0.0317 | | | 4.63 | | 0.0317 |  |
| PRS*Age at Immigration | | | | | US Born | | | 0.1150 | | | 2.30 | | 0.0576 |  |
|  | | | | | 0 < 5 | | | 0.0093 | | |  | |  |  |
|  | | | | | 6<12 | | | 0.6766 | | |  | |  |  |
|  | | | | | 13 < 21 | | | 0.6622 | | |  | |  |  |
| PRS*JAMA Healthy Diet | | | | |  | | | 0.0066 | | | 7.43 | | 0.0066 |  |
| PRS*Sleep Duration | | | | |  | | | 0.0367 | | | 4.38 | | 0.0367 |  |
|  | | | | |  | | |  | | |  | |  |  |
| ++ Omitted in next joint model | | | | | | | | | | | | | |  |
| Stage 2: (3) Adjust for multiple comparisons using these remaining 4 interactions to be included in full model with nominal significance then adjusted for multiple comparisons Bonferroni p<0.05/4=0.0125 | | | | | | | | | | | | | |  |
|  | | | | |  | | | **GxE Pr>\|t\|** | | |  | |  |  |
| PRS*Age at Immigration | | | | | US Born | | | 0.1150 | | |  | |  |  |
|  | | | | | 0 < 5 | | | 0.0093** | | |  | |  |  |
|  | | | | | 6<12 | | | 0.6766 | | |  | |  |  |
|  | | | | | 13 < 21 | | | 0.6622 | | |  | |  |  |
| PRS*JAMA Healthy Diet | | | | |  | | | 0.0066** | | |  | |  |  |
|  | | | | |  | | |  | | |  | |  |  |
|  | | | | |  | | |  | | |  | |  |  |
|  | | | | | | | | | | | | | |  |
| ****Stage 2 Interactions to be included in full model Bonferroni P(0.05/4)=0.0125** | | | | | PRS x Age at Immigration and PRS x JAMA Healthy Diet | | | | | | | | |  |
| **STAGE 3** | | | | | | | | | | | | | |  |
| Stage 3: Full Model based model building PRS*Environmental Interactions stages 1 and 2 with to be included in analysis | | | | | | | | | | | | | |  |
| **Full Model with Interactions** | | | | | BMI= Intercept + PRS + Top 5 PCs + diabetes + Sleep Duration +Cigarette Use + sex + Age^2^ + Age + Physical Activity + Cardiovascular Disease + Immigrant Generation+ Alcohol use + Sugar Sweetened Bev + Employment status + Age at Immigration + Education + JAMA Healthy Diet + Income + PRS*Age at Immigration + PRS*JAMA Healthy Diet + error | | | | | | | | |  |

| **Supplemental Table 2. HCHS/SOL Target Population Demographics for Visit 1 (2008-2011) and Visit 2 (2014-2017) unweighted and weighted** | | | | | | |
| --- | --- | --- | --- | --- | --- | --- |
|  | **Visit 1** | | | **Visit 2** | | |
|  | Unweighted Sample | Weighted Proportion | | Unweighted sample | Weighted Proportions | |
|  | n | n | Mean or %, n (std) | n | n | Mean or %, n (std) |
| **Total** | 16,415 | 16,415 | 100% | 11,623 | 11,623 |  |
| ***Sociodemographic Factors*** |  |  |  |  |  |  |
| **Age (years)** | 16,243 | 16,243 | Mean=41.05, std=0.25 | 11,623 | 11,623 | Mean=47.25, std=0.27 |
| **Gender** |  |  |  |  |  |  |
| Female | 9,740 | 8,474 | 52.2% | 7,342 | 6,057 | 52.1% |
| Male | 6,503 | 7,769 | 47.8% | 4,281 | 5,566 | 47.9% |
| **Education** |  |  |  |  |  |  |
| Less than High School | 6,207 | 5,287 | 32.3% | **--** |  |  |
| At least High School or Equivalent | 10,117 | 11,059 | 67.7% |  |  |  |
| Missing | 91 | 91 |  |  |  |  |
| **Lives with Spouse** |  |  |  | **--** |  |  |
| No | 7,979 | 8,437 | 51.4% |  |  |  |
| Yes | 8,436 | 7,978 | 48.6% |  |  |  |
| **Household Income** |  |  |  |  |  |  |
| <$30,000 USD | 10,516 | 9,994 | 60.9% | **6,317** | **5,765** | **49.6%** |
| At least $30,000 USD | 4,877 | 5,319 | 32.4% | **4,441** | **4,928** | **42.4%** |
| Missing | 1,022 | 1,101 | 6.7% | **865** | **930.3** | **8.0%** |
| **4-level employment status** |  |  |  |  |  |  |
| Retired and not currently employed | 1,545 | 1,333 | 8.3% | 2,366 | 1,732 | 15.9% |
| Not retired (or missing on retirement) and not currently employed | 6,408 | 6,588 | 41.0% | 2,464 | 2,258 | 20.7% |
| Employed part-time (<=35 hours/week) | 2,728 | 2,732 | 17.0% | 2,500 | 2,619 | 24.0% |
| Employed full-time (>35 hours/week)employed | 5,428 | 5,411 | 33.7% | 3,750 | 4,294 | 39.4% |
| Missing | 306 | 306 |  | 543 | 543 |  |
| ***Acculturation Measures*** |  |  |  |  |  |  |
| **Born in mainland U.S.** |  |  |  |  |  |  |
| Not born in 50 US States/DC | 13,479 | 12,623 | 77.2% | 9,823 | 9,002 | 77.5% |
| Born in 50 US States/DC Only | 2,863 | 3,733 | 22.8% | 1,800 | 2,621 | 22.5% |
| Missing | 73 | 73 |  | 0 | 0 |  |
| **US Born (including territories)** |  |  |  |  |  |  |
| Not born in US or US territory | 11,908 | 11,286 | 69.0% | 8,720 | 8,049 | 69.3% |
| Born in US or US territory | 4,434 | 5,070 | 31.0% | 2,903 | 3,574 | 30.7% |
| Missing | 73 | 73 |  |  |  |  |
| **Years lived in U.S.** | 16,295 | 16,295 | Mean=20.32, std=0.33 | 11,577 | 11,577 | Mean=26.24, std=0.34 |
| **Immigrant Generation** |  |  |  |  |  |  |
| 1^st^ Generation | 13,221 | 12,345 | 75.6% |  |  |  |
| 2^nd^ Generation | 3,100 | 3,991 | 24.4% |  |  |  |
| Missing | 94 |  |  |  |  |  |
| **Age at Immigration** | 13,432 | 13,432 | Mean=26.88, std=0.33 | -- | -- | -- |
| US Born | 2,863 | 3,733 | 22.8% |  |  |  |
| Under 20 | 4,146 | 4,558 | 27.9% |  |  |  |
| 20 and Over | 9,333 | 8,065 | 49.3% |  |  |  |
| Missing | 73 | 73 |  |  |  |  |
| **Background** |  |  |  |  |  |  |
| Central American | 1,321 | 973.5 | 7.8% | 921 | 714.28 | 8.0% |
| Cuban | 2,201 | 3,026 | 24.3% | 1,532 | 2,139 | 24.1% |
| Dominican | 1,155 | 1,264 | 10.1% | 817 | 894.07 | 10.1% |
| Mexican | 4,489 | 4,380 | 35.1% | 3,368 | 3,158 | 35.5% |
| Puerto Rican | 2,187 | 2,092 | 16.8% | 1,455 | 1,499 | 16.9% |
| South American | 874 | 727.9 | 5.8% | 634 | 480.19 | 5.4% |
| Missing | 4,188 | 4,188 |  | 2,896 | 2,896 |  |
| **SASH_Lang** | 16,313 | 16,313 | Mean=2.13, std=0.025 | 11,181 | 11,181 | Mean=2.18, std=0.026 |
| **SASH_Soc** | 15,686 | 15,686 | Mean=2.24, std=0.011 | 10,925 | 10,925 | Mean=2.27, std=0.012 |
| **Study Center** |  |  |  |  |  |  |
| The Bronx | 4,076 | 4,712 | 29.0% | 2,649 | 3,371 | 29.0% |
| Chicago | 4,111 | 2,577 | 15.9% | 3,089 | 1,835 | 15.8% |
| Miami | 4,017 | 4,734 | 29.1% | 2,852 | 3,401 | 29.3% |
| San Diego | 4,039 | 4,222 | 26.0% | 3,033 | 3,017 | 26.0% |
| Missing | 172 | 172 |  |  |  |  |
| ***Lifestyle Factors*** |  |  |  |  |  |  |
| **Diabetes History** |  |  |  |  |  |  |
| Non-diabetic | 13,176 | 13,956 | 85.1% | 7,737 | 8,645 | 74.4% |
| Diabetic | 3,218 | 2,440 | 14.9% | 3,882 | 2,975 | 25.6% |
| Missing | 21 | 21 |  | 4 |  |  |
| **CVD Risk Factors** |  |  |  |  |  |  |
| No prevalent CVD | 15,294 | 15,395 | 94.2% | 10,631 | 10,794 | 92.9% |
| Prevalent CVD | 1,044 | 947.9 | 5.8% | 992 | 829.3 | 7.1% |
| Missing | 77 | 77 |  |  |  |  |
| **Sugar sweetened beverages** | 16,172 | 16,172 | Mean=1.85, std=0.015 | -- | -- | -- |
| **Physical Activity** |  |  |  |  |  |  |
| Meets 2008 activity level guidelines | 5,893 | 5,374 | 33.0% |  |  |  |
| Does not meet 2008 activity level guidelines | 10,382 | 10,898 | 67.0% |  |  |  |
| Missing | 140 | 140 |  |  |  |  |
| **Language Preference** |  |  |  |  |  |  |
| Spanish | 13,119 | 12,288 | 74.9% |  |  |  |
| English | 10,382 | 4,127 | 25.1% |  |  |  |
| **Diet Score JAMA** |  |  |  |  |  |  |
| Below 60^th^ Percentile | 8,045 | 8,928 | 55.2% |  |  |  |
| Above 60^th^ Percentile | 8,127 | 7,245 | 44.8% |  |  |  |
| Missing | 243 | 243 |  |  |  |  |
| **Alternative Health Eating Index 2010** | 16,172 | 16,172 | Mean=47.45, std=0.17 | -- | -- | -- |
| **Average Sleep Duration (hrs)** | 15,530 | 15,530 | Mean=7.98, std=0.018 | -- | -- | -- |
| **Cigarette Use** |  |  |  |  |  |  |
| Never | 9,923 | 10,002 | 61.3% | 7,207 | 7,206 | 62.1% |
| Former | 3,233 | 2,832 | 17.4% | 2,665 | 2,362 | 20.4% |
| Current | 3,166 | 3,489 | 21.4% | 1,735 | 2,034 | 17.5% |
| Missing | 93 | 93 |  | 16 | 16 |  |
| **Alcohol Use** |  |  |  |  |  |  |
| No current use | 8,600 | 7,904 | 48.3% | 5,112 | 4,375 | 37.7% |
| Low level use | 6,917 | 7,451 | 45.6% | 6,043 | 6,643 | 57.3% |
| High level use | 828 | 996.7 | 6.1% | 444 | 576.3 | 5.0% |
| Missing | 70 | 70 |  | 24 | 24 |  |
|  |  |  |  |  |  |  |
| ***Psychological factors*** |  |  |  |  |  |  |
| **CES10 Summary Score** | 16,059 | 16,059 | Mean=7.00, std=0.082 | 11,181 | 11,181 | Mean=6.36, std=0.091 |
| **Ethnic Identification score** | 16,175 |  |  |  |  |  |
| **Waist to Hip Ratio** | 16,341 | 16,341 | Mean=0.92, std=0.0010 | 11,217 | 11,217 | Mean=0.93, std=0.0012 |
| **BMI (kg/m2)** | 16,344 | 16,344 | Mean=29.36, std=0.093 | 11,245 | 11,245 | Mean=29.92, std=0.10 |
| **Obesity (BMI>=30)** |  |  |  |  |  |  |
| No | 9,508 | 9,942 | 60.6% |  |  |  |
| Yes | 6,907 | 6,473 | 39.4% |  |  |  |
| **PRS Genetic Risk Score** | 10,240 | 10,240 | Mean= -0.024, std=0.015 |  |  |  |
| Unweighted Sample, Weighted proportions reflect the sampling methodology strata, PSU, and sampling weight described in Sorlie et al. Not all measures collected at both visits. | | | | | | |

| Supplemental Table 3. Weighted Interactions Polygenic Risk Score for Obesity by Body Mass Index (PRS_BMI_) and Acculturation, Sociocultural Covariates from Visit 1 (2008-2011) HCHS/SOL | | | | | | |
| --- | --- | --- | --- | --- | --- | --- |
|  | βE | P-value^a^ | βGxE^b^ | P-value^a^ | F | Pr>F, |
| Age | 0.02 | 0.0033 | 0.00 | 0.7827 | 0.08 | 0.7827 |
| Age^2^ | 0.00 | 0.0205 | 0.00 | 0.6042 | 0.27 | 0.6042 |
| Sex | 1.14 | <.0001 | 0.39 | 0.0355 | 4.44 | 0.0355* |
| HS Education | 0.37 | 0.0551 | -0.14 | 0.409 | 0.68 | 0.409 |
| Age at Immigration |  |  |  |  | 3.39 | 0.0093** |
| Born in US | 1.11 | 0.001 | 0.42 | 0.1896 |  |  |
| 0 < 5 Arrived in US | 1.61 | 0.0043 | 1.37 | 0.0022** |  |  |
| 6 < 12 Arrived in US | 0.17 | 0.728 | 0.17 | 0.728 |  |  |
| 13 <20 Arrived in US | -0.28 | 0.2067 | -0.28 | 0.2067 |  |  |
| >21 Arrived in US+ |  |  |  |  |  |  |
| Does not meet Physical Activity Guidelines | 0.86 | <.0001 | 0.21 | 0.2355 | 1.41 | 0.2355 |
| SASH Language ^C^ | 0.21 | 0.0514 | 0.21 | 0.0357* | 4.43 | 0.0357* |
| SASH Social and Ethnic Relations ^C^ | 0.12 | 0.4603 | 0.21 | 0.2122 | 1.56 | 0.2122 |
| Married living with spouse | -0.10 | 0.631 | -0.55 | 0.0076 | 7.18 | 0.0076** |
| Language Preference | 0.79 | 0.0065 | 0.51 | 0.069 | 3.32 | 0.069 |
| >30,000K Income | -0.26 | 0.217 | -0.31 | 0.1071 | 2.6 | 0.1071 |
| Employment Status |  |  |  |  | 2.58 | 0.0527 |
| Retired and not currently employed | 0.90 | 0.014 | 0.61 | 0.0866 |  |  |
| Not retired and not currently employed | 0.83 | <.0001 | 0.47 | 0.0276 |  |  |
| Employed part-time (<=35 hours/week) | -0.09 | 0.7628 | 0.32 | 0.157 |  |  |
| Employed full-time (>35 hours/week) + |  |  |  |  |  |  |
| Diabetes per ADA guideline | 2.09 | <.0001 | 0.30 | 0.1688 | 1.9 | 0.1688 |
| Prevalence CVD | 1.60 | <.0001 | 0.32 | 0.3779 | 0.78 | 0.3779 |
| Cigarette Use |  |  |  |  | 1.56 | 0.2104 |
| Former | 0.68 | 0.0032 | 0.45 | 0.1508 |  |  |
| Current | -0.75 | 0.0013 | -0.12 | 0.5891 |  |  |
| Never+ |  |  |  |  |  |  |
| Alcohol Use Drinking Level |  |  |  |  | 2.26 | 0.1056 |
| Low level use | -0.77 | 0.0001 | -0.44 | 0.0356 |  |  |
| High level use | -0.16 | 0.6861 | -0.15 | 0.6464 |  |  |
| No current use + |  |  |  |  |  |  |
| Ethnic Identity | -0.16 | 0.2514 | 0.00 | 0.9982 | 0 | 0.9982 |
| Sugar-sweetened beverages and fruit juice ^C^ servings/d | -0.29 | 0.0077 | 0.08 | 0.581 | 0.3 | 0.581 |
| Alternative Healthy Eating Index 2010 ^C^ | -0.03 | 0.0438 | -0.01 | 0.3384 | 0.92 | 0.3384 |
| JAMA Healthy Diet | -0.64 | 0.004 | -0.48 | 0.0095** | 6.76 | 0.0095** |
| Sleep Duration (hrs/day) ^C^ | -0.30 | <.0001 | -0.15 | 0.0219* | 5.28 | 0.0219* |
| Immigrant Generation | 0.29 | 0.3509 | 0.29 | 0.3509 | 0.87 | 0.3509 |
| CESD-2010 depression 10-item total summary score ^C^ | 0.06 | 0.0001 | 0.03 | 0.0683 | 3.34 | 0.0683 |
| +referent group  ^C^ survey-mean centered continuous variable  ^a^ Pr > \|t\| *p < .05, **p < .01, ***p < .001.  All Betas adjusted for top 5 principal components, study center, Hispanic/Latino background group. βE Estimates for the effect of the Environmental (Acculturation or Sociocultural) Factor on BMI (kg/m^2^). Adjusted for Interactions. | | | | | | |

| Supplemental Table 4. Weighted Interactions Polygenic Risk Score for Obesity by Body Mass Index (PRS_BMI_) and Acculturation, Sociocultural Covariates from Visit 2 (2014-2017) HCHS/SOL (n=5,781) | | | | | | |
| --- | --- | --- | --- | --- | --- | --- |
|  | βE | P-value^a^ | βGxE^b^ | P-value^a^ | F^d^ | Pr>F, |
| Sex | 1.16 | <.0001 | 0.13 | 0.4977 | 0.46 | 0.4977 |
| Age at Immigration |  |  |  |  | 2.12 | 0.0766 |
| Born in US | 1.39 | 0.0003 | 0.60 | 0.0617 |  |  |
| 0 < 5 Arrived in US | 1.79 | 0.0013 | 1.04 | 0.0248 |  |  |
| 6 < 12 Arrived in US | 1.19 | 0.0084 | 0.38 | 0.4552 |  |  |
| 13 <20 Arrived in US | -0.03 | 0.9223 | -0.05 | 0.8406 |  |  |
| >21 Arrived in US+ |  |  |  |  |  |  |
| SASH Language ^C^ | 0.33 | 0.0067 | 0.25 | 0.0166 | 5.77 | 0.0166 |
| SASH Social and Ethnic Relations ^C^ | 0.00 | 0.9984 | 0.25 | 0.1368 | 2.22 | 0.1368 |
| Married living with spouse | -0.05 | 0.8084 | -0.52 | 0.0091 | 6.85 | 0.0091 |
| Language Preference | 0.76 | 0.0134 | 0.59 | 0.0332 | 4.56 | 0.0332 |
| >30,000K Income | 0.37 | 0.0996 | 0.56 | 0.0063 | 3.79 | 0.0232 |
| Employment Status |  |  |  |  | 1.35 | 0.2582 |
| Retired and not currently employed | -0.23 | 0.4114 | 0.35 | 0.1416 |  |  |
| Not retired and not currently employed | 1.33 | <.0001 | 0.12 | 0.6722 |  |  |
| Employed part-time (<=35 hours/week) | 0.71 | 0.012 | 0.46 | 0.0959 |  |  |
| Employed full-time (>35 hours/week) + |  |  |  |  |  |  |
| Diabetes per ADA guideline | 1.55 | <.0001 | 0.20 | 0.3188 | 1 | 0.3188 |
| Prevalence CVD | 0.96 | 0.0094 | 0.23 | 0.4778 | 0.5 | 0.4778 |
| Cigarette Use |  |  |  |  | 0.83 | 0.4383 |
| Former | 0.07 | 0.7719 | -0.29 | 0.2019 |  |  |
| Current | -1.27 | <.0001 | -0.20 | 0.5188 |  |  |
| Never+ |  |  |  |  |  |  |
| Alcohol Use Drinking Level |  |  |  |  | 1.33 | 0.2649 |
| Low level use | -0.52 | 0.0202 | -0.32 | 0.105 |  |  |
| High level use | -0.42 | 0.4155 | -0.14 | 0.785 |  |  |
| No current use + |  |  |  |  |  |  |
| CESD-2010 depression 10-item total summary score ^C^ | 0.10 | <.0001 | 0.03 | 0.1309 | 2.29 | 0.1309 |
| +referent group  ^a^ Pr > \|t\| *p < .05, **p < .01, ***p < .001  b PRS _BMI_ x E  ^C^ survey-mean centered continuous variable  ^d^ F of GxE, similar to Wald F statistic  All Betas adjusted for top 5 principal components, study center, Hispanic/Latino background group. βE Estimates for the effect of the Environmental (Acculturation or Sociocultural) Factor on BMI (kg/m^2^). Adjusted for Interactions. | | | | | | |

| **Supplemental Table 5. Polygenic Risk Score (PRS) –Acculturation and Environmental Interactions for Obesity (Body Mass Index, BMI) among HCHS/SOL participants for Visit 1 (2008-2011) n=8,109^a^ Total and sex-Stratified using Alternative Healthy Eating Index 2010 as Dietary Interaction Term** | | | | | | | | | | | | | |
| --- | --- | --- | --- | --- | --- | --- | --- | --- | --- | --- | --- | --- | --- |
| Full Model | | | | | | | | | | | | | |
| Total  R^2^=0.1519 | | | |  | Males  R^2^=0.1519 | | | |  | Females  R^2^=0.1686 | | | |
| $\beta_{G}^{b}$ | SE | $\beta_{GxE}$ | SE |  | $\beta_{G}^{b}$ | SE | $\beta_{GxE}$ | SE |  | $\beta_{G}^{b}$ | SE | $\beta_{GxE}$ | SE |
|  |  |  |  |  |  |  |  |  |  |  |  |  |  |
| 1.31*** | 0.13 | 0.03^c^ | 0.19 |  | 1.26*** | 0.13 | 0.04 | 0.29 |  | 1.42*** | 0.12 | -0.19^c^ | 0.23 |
|  |  |  |  |  |  |  |  |  |  |  |  |  |  |
| All β_G^b^ P<.0001  *p < .05, **p < .01, ***p < .001.  ^a^ Analytic sample includes participants with available genetic consent for study and complete case analysis  ^b^ β_G_ for PRS_BMI_ 1 SD unit increase in PRS corresponds to kg/m^2^ change in BMI  ^C^ PRS_BMI_ x Diet using Alternative Healthy Eating Index 2010 dichotomized by top 40% percentile by sex  All models account for HCHS/SOL complex survey design and sampling weights adjusted for Environmental € factors which explained variability based on Visit 1 backwards elimination inferential building. | | | | | | | | | | | | | |

| **Supplemental Table 6**. Weighted Number of HCHS/SOL participants in each obesity polygenic risk score (PRS) quantile by Baseline BMI category (normal weight: 18.5≤BMI<25; overweight: 25≤BMI<30; non-severely obese: 30≤BMI<40; severely obese: BMI≥40). | | | | | | | | | | | | | | | |
| --- | --- | --- | --- | --- | --- | --- | --- | --- | --- | --- | --- | --- | --- | --- | --- |
|  | **Normal Weight** | | |  | **Overweight** | | |  | **Non-Severely Obese** | | |  | **Severely Obese** | | |
| **PRS** | Visit 1  (n, %) | Visit 2 (n, %) | Change |  | Visit 1  (n, %) | Visit 2 (n, %) | Change |  | Visit 1  (n, %) | Visit 2 (n, %) | Change |  | Visit 1(n, %) | Visit 2 (n, %) | Change |
| **Mean** | 22.9 | 23.7 | 0.03 |  | 27.6 | 27.6 | 0 |  | 33.5 | 32.9 | -0.02 |  | 44.9 | 42.2 | -0.06 |
| **Percentile^a^** | 264 | 159 | -0.40 |  | 361 | 256 | -0.29 |  | 177 | 139 | -0.21 |  | 12 | 8 | -0.33 |
| **10th** | 213 | 129 | -0.39 |  | 352 | 256 | -0.27 |  | 229 | 157 | -0.31 |  | 27 | 19 | -0.30 |
| **20th** | 164 | 106 | -0.35 |  | 353 | 229 | -0.35 |  | 256 | 173 | -0.32 |  | 32 | 26 | -0.19 |
| **30th** | 155 | 100 | -0.35 |  | 337 | 232 | -0.31 |  | 300 | 205 | -0.32 |  | 32 | 26 | -0.19 |
| **40th** | 158 | 101 | -0.36 |  | 327 | 216 | -0.34 |  | 299 | 180 | -0.40 |  | 40 | 29 | -0.28 |
| **50th** | 132 | 83 | -0.37 |  | 334 | 223 | -0.33 |  | 310 | 218 | -0.30 |  | 46 | 38 | -0.17 |
| **60th** | 117 | 52 | -0.56 |  | 304 | 218 | -0.28 |  | 250 | 253 | 0.01 |  | 53 | 46 | -0.13 |
| **70th** | 91 | 57 | -0.37 |  | 308 | 215 | -0.30 |  | 362 | 253 | -0.30 |  | 61 | 38 | -0.38 |
| **80th** | 77 | 43 | -0.44 |  | 277 | 189 | -0.32 |  | 390 | 265 | -0.32 |  | 86 | 51 | -0.41 |
| **90th** | 65 | 38 | -0.42 |  | 259 | 176 | -0.32 |  | 381 | 252 | -0.34 |  | 111 | 64 | -0.42 |
| ^a^PRS percentile cut offs based on Visit 1: 10^th^:-1.34, 20^Th^ -0.86, 30^th^ -0.53, 40^th^ -0.23, 50^th^ -0.02, 60th 0.25, 70^th^ 0.50, 80^th^ 0.81, 90^th^ 1.24, 4.06  BMI Categories: BMI category (normal weight: 18.5≤BMI<25; overweight: 25≤BMI<30; non-severely obese: 30≤BMI<40; severely obese: BMI≥40). | | | | | | | | | | | | | | | |

| **Supplemental Table 7. BMI kg/m2 Across Both Visit 1 and Visit 2 HCHS/SOL** | | | |
| --- | --- | --- | --- |
|  | **Total** | **Men** | **Women** |
| **Visit 1 (n=8,109)** | **29.8 (0.10)** | **29.9 (0.12)** | **30.4 (0.14)** |
| **Visit 2 (n-5,781)** | **29.9 (0.10)** | **29.4 (0.14)** | **30.6 (0.17)** |
